# Morphological and Genomic Characterization of *Filobasidiella depauperata*: A Homothallic Sibling Species of the Pathogenic *Cryptococcus* Species Complex

**DOI:** 10.1371/journal.pone.0009620

**Published:** 2010-03-10

**Authors:** Marianela Rodriguez-Carres, Keisha Findley, Sheng Sun, Fred S. Dietrich, Joseph Heitman

**Affiliations:** Department of Molecular Genetics and Microbiology, Duke University Medical Center, Durham, North Carolina, United States of America; Massachusetts General Hospital, United States of America

## Abstract

The fungal species *Cryptococcus neoformans* and *Cryptococcus gattii* cause respiratory and neurological disease in animals and humans following inhalation of basidiospores or desiccated yeast cells from the environment. Sexual reproduction in *C. neoformans* and *C. gattii* is controlled by a bipolar system in which a single mating type locus (*MAT*) specifies compatibility. These two species are dimorphic, growing as yeast in the asexual stage, and producing hyphae, basidia, and basidiospores during the sexual stage. In contrast, *Filobasidiella depauperata*, one of the closest related species, grows exclusively as hyphae and it is found in association with decaying insects. Examination of two available strains of *F. depauperata* showed that the life cycle of this fungal species shares features associated with the unisexual or same-sex mating cycle in *C. neoformans*. Therefore, *F. depauperata* may represent a homothallic and possibly an obligately sexual fungal species. RAPD genotyping of 39 randomly isolated progeny from isolate CBS7855 revealed a new genotype pattern in one of the isolated basidiospores progeny, therefore suggesting that the homothallic cycle in *F. depauperata* could lead to the emergence of new genotypes. Phylogenetic analyses of genes linked to *MAT* in *C. neoformans* indicated that two of these genes in *F. depauperata*, *MYO2* and *STE20*, appear to form a monophyletic clade with the *MAT* **a** alleles of *C. neoformans* and *C. gattii*, and thus these genes may have been recruited to the *MAT* locus before *F. depauperata* diverged. Furthermore, the ancestral *MAT* **a** locus may have undergone accelerated evolution prior to the divergence of the pathogenic *Cryptococcus* species since several of the genes linked to the *MAT* **a** locus appear to have a higher number of changes and substitutions than their *MAT*α counterparts. Synteny analyses between *C. neoformans* and *F. depauperata* showed that genomic regions on other chromosomes displayed conserved gene order. In contrast, the genes linked to the *MAT* locus of *C. neoformans* showed a higher number of chromosomal translocations in the genome of *F. depauperata*. We therefore propose that chromosomal rearrangements appear to be a major force driving speciation and sexual divergence in these closely related pathogenic and saprobic species.

## Introduction

Previous studies have shown that selective forces promote clustering of genes that are co-expressed because they are involved in the same biosynthetic pathway or developmental process. However, the genomic events contributing to the rearrangement and maintenance of gene clusters are less well understood [1]. The genomic arrangements of sex chromosomes present an interesting example of such mechanisms, particularly since convergent evolutionary events appear to have directed the formation of sex determining regions in fungi and animals. For example, it has been postulated that the sex-determining locus of the human pathogenic fungi *C. neoformans* and *C. gattii* were shaped by genomic events similar to those that drove the evolution of sex chromosomes in mammals, including sequential gene acquisitions, suppression of recombination, and chromosomal rearrangements [2].

In fungi, sexual reproduction is determined by compatibility at the *MAT* locus. In many basidiomycetes mating is controlled by a tetrapolar system in which two unlinked loci regulate recognition and sexual compatibility [3]–[5]. For mating to occur, the interacting partners must have different alleles at both loci. One locus encodes pheromones and pheromone receptors while the other encodes homeodomain proteins. However, in the basidiomycetous yeasts *C. neoformans* and *C. gattii* mating is controlled by a bipolar mating system in which a single *MAT* locus contains the pheromone/pheromone receptor genes linked to the genes encoding the homeodomain proteins [6]. Under laboratory conditions sexual reproduction is triggered in these fungi by the recognition of a compatible mating partner that usually carries a different *MAT* allele (*MAT*α and *MAT* **a**). Fusion of compatible haploid cells produces an extended and free-living dikaryotic hyphae with clamp connections. The dikaryotic hyphae differentiate into basidia, sac-shaped sexual structures where karyogamy and meiosis occur, resulting in the production of haploid spores called basidiospores. Basidiospores subsequently germinate into yeast cells that divide mitotically by budding, initiating the asexual stage of the life cycle.

In *C. neoformans* a modified version of this sexual cycle, called monokaryotic fruiting, also occurs under laboratory conditions and in nature. Monokaryotic fruiting involves self-fertility, also known as same-sex mating [7]–[8]. During this process, monokaryotic hyphae with unfused clamp connections are formed, which then give rise to basidia followed by the production of haploid basidiospores [7]–[9]. Disruption of genes involved in meiosis and recombination severely compromises spore production and viability, and analysis of recombination in basidiospores produced by this process demonstrated that monokaryotic fruiting is a form of homothallism, or self-fertility [7].

*C. neoformans* and *C. gattii* are opportunistic human pathogens that are ubiquitous in the environment. They are commonly isolated from avian guano and trees, and less frequently they have been found in decaying wood, vegetables, insects, and soil [6]. Same-sex mating is thought to have contributed to the generation of the hypervirulent genotype of *C. gattii* that is causing the cryptococcosis outbreak on Vancouver Island [10], and α/α (alpha/alpha) diploids in the *C. neoformans* population [11]. Although population analyses of clinical and environmental isolates have revealed the existence of a wide range of genotypes, one mating type, the α-mating type allele (*MAT*α) is overwhelmingly predominant in clinical and environmental isolates. The α allele has also been associated with higher virulence in certain genetic backgrounds and modified forms of the sexual cycle that involve same-sex mating [6]. Therefore, the complexities of sexual cycle transitions, as well as the structure of *MAT* are thought to be fundamental to our understanding of the pathogenic potential of these fungi.

In *C. neoformans* and *C. gattii* the *MAT* loci are large (>100 kb) and contain many clustered genes, several of which play a role during mating [2]. Phylogenetic analysis of these *MAT*-associated genes in *Cryptococcus* revealed two main evolutionary lineages. The majority of the genes within the *MAT* locus (*STE12, STE3, IKS, MYO2, STE20, ZNF1, PRT1, BSP3, ETF1, RPL39, RPL22, SPO14, RUM1*, & *BSP1*) show a mating-type specific topology, while a few genes (*GEF1, LPD1, CID1, RPO41,* & *BSP2*) reflect a species-specific phylogeny [2]. Therefore, Fraser and colleagues proposed a series of events that led to the formation of this bipolar mating system: a) several rounds of sequential gene acquisitions expanding the pheromone/pheromone receptor locus and the homeodomain region, b) a chromosomal translocation that linked these two regions into a contiguous one resulting in a transitional tripolar intermediate, c) resolution of this transitional tripolar state via gene conversion or recombination that gave rise to the current bipolar system in *Cryptococcus*. Evidence supporting an ancestral tetrapolar system was provided by the creation of stable and fertile tripolar and tetrapolar laboratory strains of *C. neoformans* [12]. Recent findings suggest that these genomic events shaping the evolution of the *MAT* locus in pathogenic *Cryptococcus* species might have occurred prior to their speciation. The majority of the *MAT*-associated genes in the bipolar *Cryptococcus* species are found as two separate clusters in the genomes of two closely related tetrapolar yeasts in the Tremellales, *Tremella mesenterica* and *Cryptococcus heveanensi*s [Metin et al. *in prep.*].

*C. neoformans* and *C. gattii* were classified into their own teleomorphic genus, *Filobasidiella*, based on the presence of morphological features unique to their sexual cycle. For example, the basidia of most *Tremella* species, and its close relatives, are vertically septated; however, the basidia of *C. neoformans* and *C. gattii* are non-segmented [13]. Another distinguishing characteristic is the presence of four basipedal spore chains on the basidium of *C. neoformans* and *C. gattii*. Recently, phylogenetic studies based on six conserved loci confirmed that *C. neoformans* and *C. gattii* form a monophyletic lineage within the Tremellales [14]. According to Findley et al. three additional species, *Cryptococcus amylolentus*, *Tsuchiyaea wingfieldii*, and *Filobasidiella depauperata*, cluster within the *Filobasidiella* clade. These three species are all found in similar habitats (decaying insect or insect frass) as those previously reported for the pathogenic *Cryptococcus* species. Interestingly, while *C. amylolentus* and *T. wingfieldii* have been described as yeasts, *F. depauperata* appears to be exclusively filamentous. The taxonomic placement of the filamentous species *F. depauperata* within *Filobasidiella* is in agreement with its previous classification based on its basidia and basidiospore morphology [15]–[16] and molecular data from coding and non-coding regions of rDNA [17]–[19]. Although *F. depauperata* hyphae appear to lack clamp connections [15]–[16], a characteristic feature of the dikaryotic hyphae present in *C. neoformans* and *C. gattii*, evidence for its sexual stage was provided by transmission electron microscopy (TEM) images showing the presence of synaptonemal complexes in the basidia [15]. Therefore, *F. depauperata* appears to exist only in its sexual stage as a homothallic fungus, although it might occupy similar habitats to those of its saprobic and pathogenic yeast relatives.

Given the medical relevance of some members of the *Filobasidiella* clade, to gain further insight into life cycle transitions and species divergence, we have compared molecular data and morphological characteristics of the exclusively filamentous fungus *F. depauperata* and the dimorphic, pathogenic *Cryptococcus* species. Results from these studies suggest that the obligate sexual and homothallic life cycle of *F. depauperata* could generate genetic diversity. Phylogenetic and synteny analyses of five genomic regions from *F. depauperata* and *C. neoformans* revealed that chromosomal translocations are a major mechanism driving the evolution of *MAT*-associated genes, a gene cluster linked to sexual reproduction and virulence in the pathogenic *Cryptococcus* species.

## Materials and Methods

### Cultures and Media

The two strains of *F. depauperata* used in this study, CBS7855 and CBS7841, were obtained from the CBS Culture Collection, while the *C. neoformans* reference strains JEC21, H99, and XL1549 diploid control were from the Heitman lab strain collection. Strains were grown on yeast extract-peptone-dextrose (YPD) and yeast-nitrogen-base (YNB) medium. Mating assays were performed on V8 medium at pH = 5 and pH = 7 [20] and MS medium, pH = 4 [21]. Spore suspensions were made by rinsing an actively growing culture on YPD agar media with 3 ml of water. To isolate single spores, an agar fragment from the plate was excised, and transferred to a fresh plate containing YPD media for micro-dissection.

### Light and Fluorescence Microscopy

Slides coated with a thin layer of YPD agar medium were inoculated with a spore suspension from strains CBS7855 and CBS7841. Slides were then transferred to a Petri dish and incubated at room temperature under sterile conditions. Samples were grown on slides for up to seven days, and light microscopy observations were conducted every 24 hours. Before each observation, slides were washed three times with 3 ml of phosphate-buffered saline solution (PBS). Fungal cell walls were stained by submerging samples in a solution of calcoflour white (fluorescent brightener 28 F-3397; Sigma) for 10 minutes. Slides were then rinsed gently with PBS and submerged into a fixing solution (3.7% Formaldehyde and 1% Triton in PBS) for 10 minutes to permeabilize fungal tissue for subsequent staining of nucleic acids with Sytox Green (Molecular Probes) for 20 min. Slides were then rinsed with PBS two times and observed under UV light.

### Scanning Electron Microscopy (SEM)

Specimens for SEM were prepared by excising thin agar pieces from an actively growing culture and viewed on a Philips XL30 SEM TMP (FEI Company, Hillsboro, OR). For higher resolution SEM, intact agar plugs were excised from the agar slide and fixed in 3% glutaraldehyde in 0.1 M sodium cacodylate buffer, pH = 6.8 for several days at 4°C. The agar plugs were then rinsed in three 1-hour changes of cold 0.1 M sodium cacodylate buffer, pH = 6.8 followed by a graded dehydration series of 1-hour changes in cold 30% ethanol, followed by 50% ethanol, and held overnight in 70% ethanol. Dehydration was completed with 1-hour changes of cold 95% and 100% ethanol at 4°C warming to room temperature in 100% ethanol. Two additional 1-hour changes of room temperature 100% ethanol completed the dehydration series. The samples were then critical point dried in liquid CO_2_ (Samdri-795, Tousimis Research Corp., Rockville MD) for 15 minutes. The agar pieces were mounted on stubs with double stick tape, pressed down completely around the edge and then sealed with silver paint to ensure good conductivity. The samples were sputter coated with 50 Å of Au/Pd (Hummer 6.2, Anatech U.S.A., Hayward CA). Samples were held in the vacuum desiccator until viewed on a JEOL JSM 5900LV (JEOL U.S.A., Peabody, MA) SEM at 15 kV.

### Transmission Electron Microscopy (TEM)

Agar pieces were excised from cultures and fixed in a buffered solution containing 3% glutaraldehyde in 0.1 M sodium cacodylate buffer at pH = 6.8. Samples were incubated at 4°C for several days and rinsed three times for 15-minutes in buffer and then post-fixed in 2% osmium tetroxide in 0.1 M sodium cacodylate buffer, pH = 6.8 for 2 hours at 4°C. Samples were washed in 3 changes of cold buffer as above and the sample was then incubated at 60°C in a 2% agarose solution in 0.1 M sodium cacodylate buffer pH 6.8. Two additional drops of warm agarose were added, the pellet and agarose were mixed with a stick, covered with a few more drops of agarose, and then the tubes were spun for 5 minutes. The pellet containing the cells was removed and divided into 1 mm^3^ blocks in a Petri dish of distilled water. The blocks were dehydrated in a graded series (30, 50, 70, 95, 100%) of ethanol, warmed to room temperature, and infiltrated with Spurr's resin in a 1∶1 Spurr's: ethanol and a 3∶1 Spurr's: ethanol ratio and incubated overnight. Samples were infiltrated with 3 changes of 100% Spurr's under a vacuum over several days, followed by embedding in BEEM capsules with fresh 100% Spurr's overnight at 70°C. One block from each sample was hand-trimmed and then thin-sectioned using an LKB Nova ultramicrotome (Leica Microsystems, Bannockburn, IL) and a Diatome diamond knife (Electron Microscopy Sciences, Hatfield, PA); sections were collected on four 200-mesh grids. Two grids per sample were stained with 4% aqueous uranyl acetate in the dark at room temperature followed by 3 distilled water washes (slightly warmed) followed by 4 minutes in Reynold's lead citrate and 3 more distilled water washes as above. Grids were viewed using a JEOL JEM-100S (JEOL U.S.A., Peabody, MA), imaged with Kodak Type 4489 film (Eastman Kodak Co., Rochester, N.Y.), and scanned using an Epson Perfection 4870 Photo scanner (Seiko Epson Corp., Long Beach, CA) at 1200 dpi.

### DNA Extractions

Fungal DNA was extracted from a three-week old culture grown in YPD liquid at 24°C. The mycelial mat was collected by filtration lyophilized, and DNA extracted using the CTAB method [22]. Bacterial DNA was isolated using QIAGEN plasmid purification kit according to the manufacturer's instructions. Plasmid DNA from TOPO clones were extracted using the QIAprep Spin Miniprep kit (QIAGEN), fosmid DNA was extracted using the Plasmid Midi Kit (QIAGEN), and DNA from shot-gun libraries were extracted using the DirectPep 96 MiniPrep (QIAGEN).

### RAPD Genotyping of Both Strains of *F. depauperata* and Progeny from Isolate CBS7855

To detect the differences between the two isolates of *F. depauperata*, CBS7841 and CBS7855, and among the progeny of CBS7855, PCR reactions using a set of 12 random primers (Table shown in supporting File S1) were conducted. Strain ATCC36983 corresponds to CBS7841, and was used as a control for reproducibility of RAPD genotyping. Primers were designed by randomly choosing a string of 15 or 16 digits from the irrational numbers Pi, square root of 2, and constant E, and then transferring these into DNA sequences by substituting: 1 and 5 to A, 2 and 6 to T, 3, 7 and 9 to G, and 4, 8 and 0 to C. Thermal cycles were as follow: initial denature step at 94°C for 6 minutes, followed by 36 cycles of 45 seconds at 94°C, 45 seconds at the specific annealing temperature of each primer (supporting File S1), and 90 seconds at 72°C. Then the final extension step was carried out at 72°C for 7 minutes. PCR products were analyzed by agarose gel electrophoresis. The consistency of the PCR reactions and reproducibility of the unique bands observed in the progeny of CBS7855 was confirmed by repeating the PCR reaction four times and sequencing the amplified unique PCR product in the progeny.

### PCR Amplifications

PCR amplification for two highly conserved fungal genes encoding the largest subunit of RNA polymerase II (*RPB1*) and the elongation factor 1 alpha (*EF1*α) were performed according to previously published protocols [14]. PCR primer sequences and conditions for the *CAP1, STE11*, and *RUM1* genes were performed according to the methods described by Metin et al. 2009 (in prep.) for the fungus *Cryptococcus heveanensis*. Primers and conditions for touch-down PCR for the *STE20* genes were performed according to those previously used for cloning the *STE20* genes in *C. neoformans* [23]. Degenerate primers for *LPD1* (pair JOHE15841 GCCTCAAGACCGCCTGCRTNGARAARMG, and JOHE15845 GGAGGGGATGGCGGCRTARTTNACRT; pair JOHE15842 TCCGAGCCCACCCCNTTYCCNGG and JOHE15846 GGCGGCGTAGTTGACGTGNCCRTGCC), and for *NOG2* (JOHE15836 TCCAAGGAGTACCCCACCATNGCNTTYCAYG and JOHE15840 GGGATCTTGCCGCGGWTVMARTCRTT) were designed using the online computer program, COnsensus-DEgenerate Hybrid Oligonucleotide Primer (CODEHOP, http://blocks.fhcrc.org/codehop.html) from the gene alignment of *C. neoformans*, *U. maydi*s, and *C. cinereus*. PCR products were separated by gel electrophoresis and PCR products of the expected size were gel purified using the QIAquick Gel Extraction Kit (Qiagen, Valenica, CA). Products were then cloned using the TOPO-TA Cloning Kit (Invitrogen). Clones were transformed into *E. coli* for plasmid amplification and sequencing.

### Fosmid Library and Hybridizations

Fosmid libraries were constructed using the CopyControl™ Fosmid Library Production Kit (Epicentre, Madison, Wisconsin). Genomic DNA from *F. depauperata* strain CBS7855 was sheared by pipetting up and down 100 times and end-repaired. To obtain the desired fragment size (40 kb fragments) DNA was separated using Contour-Clamped Homogenous Electric Field (CHEF) electrophoresis on a CHEF DR-II apparatus (Bio-Rad, Hercules, California) under the following conditions: 1- to 6-sec switch time, 6 V/cm, 14°C for 14-15 hrs. The desired size range was excised from the gel and purified by gel extraction and precipitation. The DNA was then ligated into the CopyControl pCC1FOS cloning-ready vector. The ligated DNA was packaged into *E. coli* Phage-resistant cells according to the manufacturer's instructions. A total of ∼16,000 clones were picked into 96-well plates and replicated to 384-well plates for storage at −80°C. 384-well plates were replicated onto nitrocellulose filter papers according to the protocols described by Sambrook et al, and hybridizations were carried out in the ULTRAhyb (Ambion, Inc.) hybridizing solution. Membranes were incubated at 50°C for 10–12 h for hybridization, and subsequently washed three times at 50°C with 2X SSC, 0.1% SDS, and twice with 0.1X SSC, 0.1% SDS at 65°C.

### Sequencing and Annotation

Plasmid DNA from TOPO clones and from the shot-gun sequencing libraries made from the fosmid clones were sequenced using the M13 forward and M13 reverse universal primers. Sequencing reactions were performed using Big Dye chemistry v3.1 (Applied Biosystems, Foster City, California, United States) and analyzed on an Applied Biosystems 3730xl capillary sequencer. Sequence reads were assembled with the sequence assembly software CONSED [24] into 5 contigs. Assembled sequences were subsequently annotated employing the gene predictor software FGENESH from SoftBerry© and using as reference current gene predictions in *C. neoformans* (http://linux1.softberry.com/berry.phtml?topic=fgenesh&group=programs&subgroup=gfind). Sequences were deposited into GenBank using the stand-alone software tool Sequin (http://www.ncbi.nlm.nih.gov/Sequin/QuickGuide/sequin.htm). GenBank accession numbers for fosmids clones are as follow: for 6B03 is GU131348; for 7G23 is GU131351; 7B24 is GU131350; for 8J20 is GU131347; and for 5G13, 7D18 & 8P15 is GU131349. GenBank accession numbers for fragments of the *EF1*α gene is GU131345, and the *RPB1* gene is GU131346. The GenBank accession number for the ITS regions of strain CBS7855 is GU289923 and for the unique band in progeny 33 of CBS7855 is GU289556.

### Phylogenetic Analyses

DNA sequences were aligned using ClustalW 1.81 [25]. The FASTA alignment files for each of the genes were imported into MacClade 4.08 [26] for manual editing. Heuristic searches for maximum parsimony (MP) and maximum likelihood criteria were conducted using PAUP 4.0 [27]. Parameters for ML searches were estimated using MODELTEST [28] and statistical support was calculated from 1,000 bootstrap replicates.

### Synteny Analyses

To compare the genomes of *F. depauperata* and *C. neoformans* var. *neoformans*, a total of 7 fosmid clones (6B03, 5G13, 7G23, 7B24, 7D18, 8J20, and 8P15) from a genomic library of *F. depauperata* strain CBS7855 were sequenced, assembled and annotated. Four fosmids were selected for sequencing after probing the *F. depauperata* genomic library with several of the genes (*STE20, MYO2, LPD1, CID1*, and *STE11*) associated with the *MAT* locus of the pathogenic *Cryptococcus* species. Fosmid 8P15 was positive for the *CID1* and *MYO2* genes, fosmid 7D18 was positive for the *MYO2*, *STE20*, and *LPD1* genes, and fosmid 7B24 was positive for the *STE11* gene. Three additional randomly selected fosmids (7G23, 6B03, and 8J20) were also sequenced. Assembled contigs were subsequently aligned using as a reference the genome of *C. neoformans*, JEC21. Homologous regions between *F. depauperata* and *C. neoformans* were analyzed using GRIMM-Synteny software (http://grimm.ucsd.edu/GRIMM/) to predict the minimum number of genomic rearrangements (translocations, fusions and inversion) that might have taken place [29]–[30]. To further evaluate and compare gene arrangements, a synteny score was assigned to each of the 66 ORFs identified in *F. depauperata* and present in *C. neoformans*. The maximum synteny score ( = 2) was assigned to those ORFs fully syntenic as they were located between two adjacently conserved genes, while ORFs with only one adjacent gene was conserved were given a lower score ( = 1). Those ORFs with no adjacent genes conserved between the two species were given the lowest synteny score ( = 0).

## Results

### Phylogenetic Analysis of *F. depauperata*

**Figure 1:**
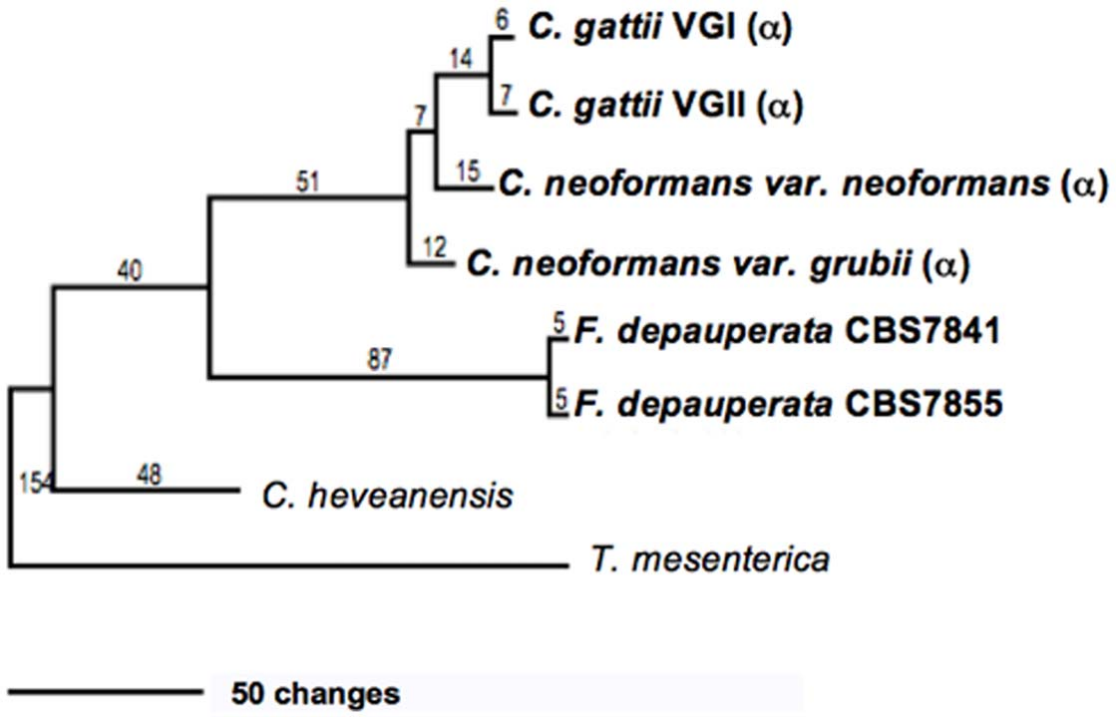
Phylogeny of *F. depauperata* and closely related species. A Maximum Parsimony tree depicting the relationship between both strains of *F. depauperata* and the pathogenic *Cryptococcus* species using a concatenated data set derived from two coding genes. Numbers above the branches indicate changes. Statistical support was calculated from 1,000 bootstrap replicates. Bootstrap values were >70% in all branches (values not shown). The “α” indicates strains with the *MAT*α locus. Unrooted trees for Maximum Parsimony and Maximum likelihood are shown in supporting File S1.

To resolve the relationship of the two available strains of *F. depauperata*, three conserved loci ITS, *EF1*α and *RPB1*, from CBS7855 were compared to publicly available sequences for strain CBS7841. Sequence comparison of these two genes between these two *F. depauperata* strains (CBS7855 and CBS7841) revealed ∼98% sequence identity. The 2% sequence differences between the *EF1*α and *RPB1* genes in *F. depauperata* appear to be the result of transitions and synonymous substitutions, consistent with recent divergence of the two strains (alignment of coding regions shown in supporting File S1). Genes from *F. depauperata* also showed ∼80% DNA sequence identity when individually compared to their counterparts in *C. neoformans* or *C. gattii* (data not shown). These results are reflected in the phylogeny derived from the two-gene concatenated data set (Figure 1). Comparison of the same conserved genes between the sequences of two isolates of *C. gattii* that belong to two different molecular groups (VGI and VGII) also displayed 98% sequence identity further supporting that the two strains of *F. depauperata* could represent cryptic species resembling the relationship of the different molecular groups present in *C. gattii* (total number of changes shown in Figure 1, unrooted trees for maximum parsimony and maximum likelihood are shown in supporting File S1).

### Genetic Similarity between Both Isolates of *F. depauperata* and among the Progeny of Isolate CBS7855

**Figure 2:**
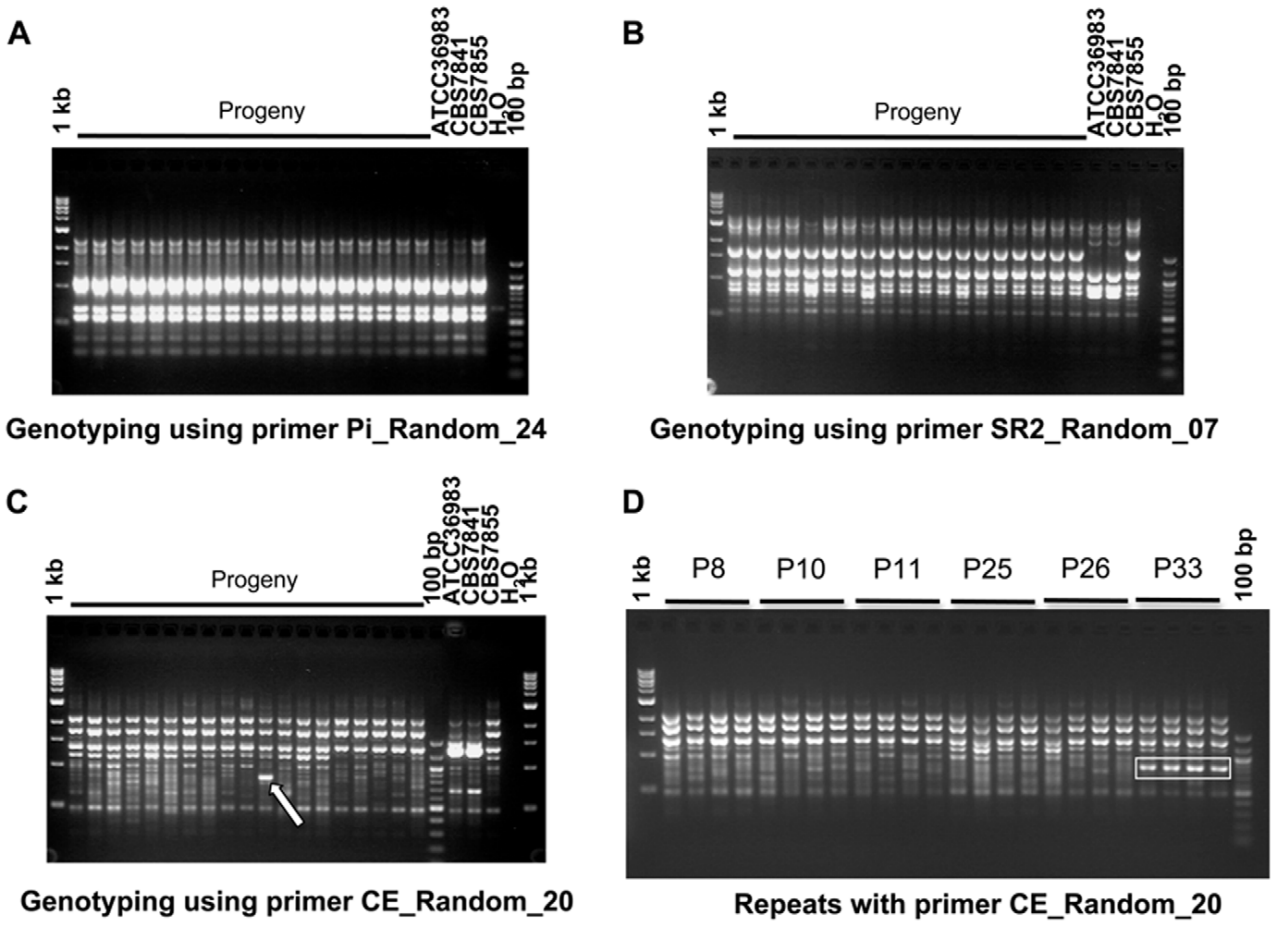
Genetic diversity between isolates of *F. depauperata* and among the progeny of isolate CBS7855. Panels A, B and C shows representative results from three of the 12 different random primers that were used to evaluate the genetic similarity of both strains of *F. depauperata*, and progeny from CBS7855. Strains CBS7841 and ATCC36983 represent the same isolate, but obtained from two different culture collections (ATCC and CBS). A total of 39 progeny were tested, but only 19 are shown in panels A, B & C. Panel A shows the results from one of the random primers (Pi_Random_24) that generated an identical PCR pattern for all isolates (CBS7841, CBS7855, and also among the progeny from CBS7855). Panel B and C shows the results from the two random primers (SR2_Random_07, and CE_Random_20) that displayed different PCR patterns for CBS7841 and CBS7855. Primer CE_Random_20 also revealed a unique band (white arrow) present in progeny #33 (P33), but not present in the parental strain CBS7855. Panel D shows the consistency and reproducibility of the unique band observed in P33 by independent repetitions of the PCR experiments. Rectangles indicated the unique PCR band that is present P33 but absent from the parental strain and other progeny. P8, P10, P11, P25, P26, and P33 correspond to progeny of CBS7855. 1 kb∶1 kb DNA ladder; 100 bp∶100 bp DNA ladder; H_2_O: negative control for the PCR reaction.

To compare the genetic similarity between the two isolates of *F. depauperata*, CBS7841 and CBS7855, and among the progeny of CBS7855, each isolate was individually genotyped using a set of 12 random primers for RAPD analyses (Table shown in supporting File S1). As expected, CBS7841 and ATCC36983, which correspond to the same isolate but obtained from two different strain collections (ATCC and CBS), had the pattern for all of the 12 random primers tested (Figure 2A, and Table shown in supporting File S1). Two (SR2_Random_07, and CE_Random_20) of the ten primers that gave unique patterns for CBS7841/ATCC36983 and CBS7855 are shown in Figure 2B and 2C. These results indicate that these two isolates are genetically distinct, and could represent different population of the same species, *F. depauperata*.

A total of 39 basidiospores from CBS7855 were isolated and grown in order to evaluate their morphological and genetic similarities. All progeny appear to be morphologically identical to the parental strain, CBS7855. At the genetic level RAPD analyses showed an identical pattern for all progeny, except P33 (progeny #33). For P33 a unique band was identified, and this band was not present in the parental strain CBS7855 (Figure 2C & 2D). These results suggest that genetic diversity could be generated through homothallic sexual reproduction.

### Growth Response to Environmental Cues

The two available strains of *F. depauperata*, CBS7841 and CBS7855, were grown under a range of conditions to characterize and compare their phenotypes and morphologies. The same inoculum size was used to start the cultures, and after 14 days the mycelial growth of isolate CBS7855 was consistently of a smaller than CBS7841 (supporting File S1). Evaluation of nutritional requirements, and sensitivity to antifungal agents (5-fluoroorotic acid, cyclosporine A, FK506, neomycin, nourseothricin, hygromycin, and rapamycin) revealed no additional distinguishing physiological characteristics between the strains, other than the apparent difference in growth (supporting File S1). Basidiospore germination and viability for each strain was determined to assess whether the observed difference in growth is due to spore germination. To determine viability spore solutions were plated onto YPD medium, and individual basidiospores were dissected. Microscopic observation of the plated and dissected spores after 48–72 hours showed similar germination and viability (80–90%) for both strains (data not shown). Yeast growth was not observed under any of the conditions tested, including different temperatures, carbon dioxide levels, low nutrients, liquid and solid media (data not shown), and therefore *F. depauperata* appears to be strictly filamentous.

**Figure 3 pone-0009620-g003:**
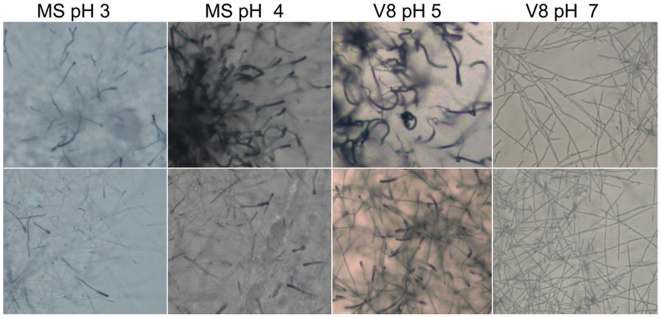
Fruiting and filamentation of *F. depauperata* at different pHs. Colony-edges (4× magnification) of *F. depauperata* growing on different types of mating media and at different pH (MS pH = 3, MS pH = 4, V8 pH = 5, and V8 pH = 7) are shown. Top panels are of strain CBS7841, and bottom panels strain CBS7855. Photographs were taken after 7 days.

Since *F. depauperata* filamentous growth mimics sexual development in *C. neoformans* and *C. gattii*, both isolates of *F. depauperata* were grown under conditions known to impact mating of *C. neoformans*. For example, under laboratory conditions nutrient rich media, high levels of CO_2_, and exposure to light have been shown to inhibit mating of *C. neoformans* [31]–[33]; however, none of these environmental cues impacted fruiting of *F. depauperata* (data not shown). The most dramatic effect observed on *F. depauperata* was caused by changes in pH (Figure 3). Numerous basidia and longer basidiospores chains were observed in both strains after 6 days at pH = 5, which is the same pH mating preference displayed by *C. neoformans* var. *grubii*. Hyphae with few basidia were observed in *F. depauperata* cultures grown at a higher or lower pH. However, after extended incubation (12 days), at pH = 7, the hyphal tips began differentiating into basidia, and a few days later basidiospores were observed.

### Morphological Examination of Basidium, Basidiospores, and Hyphal Morphology

**Figure 4 pone-0009620-g004:**
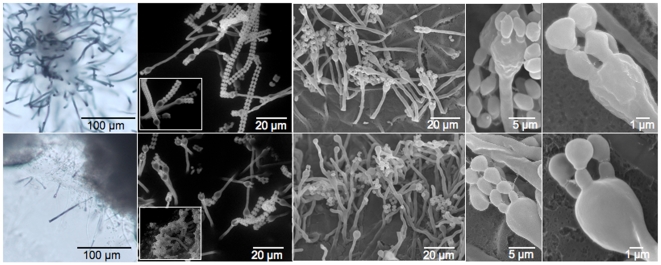
Basidia and basidiospores morphology differ between the two strains of *F. depauperata*. SEM images show that CBS7841 basidia have a rough texture, while the basidia of CBS7855 are smooth. Longer spore chains were also observed on the basidia of CBS7841. Surface field views of the colony-edges were examined by SEM. Top panel shows strain CBS7841 and bottom panel CBS7855.

**Figure 5:**
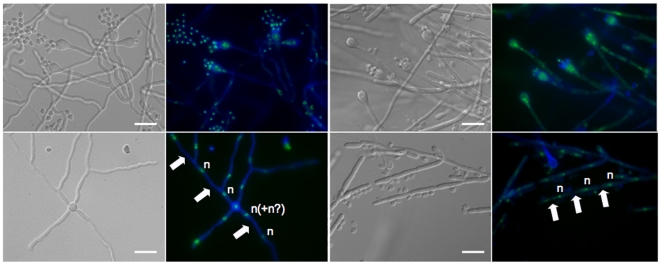
Monokaryotic hyphae, basidia, and basidiospores of *F. depauperata*. Hyphae and basidiospores from both strains of *F. depauperata* appear to be mostly monokaryotic with hyphae that lack clamp connections. Nuclear content was determined by examination under fluorescence light microscopy (color images). Samples were stained with sytox-green to detect nucleic acids (shown in green), and with calcofluor white to visualize the cell walls (shown in blue). DIC images are shown in grey. White arrows indicate cell wall septa, and the letter “n” represents the observed nuclear content. The two left panels are of strain CBS7841, and the two right panels are CBS7855.

**Figure 6:**
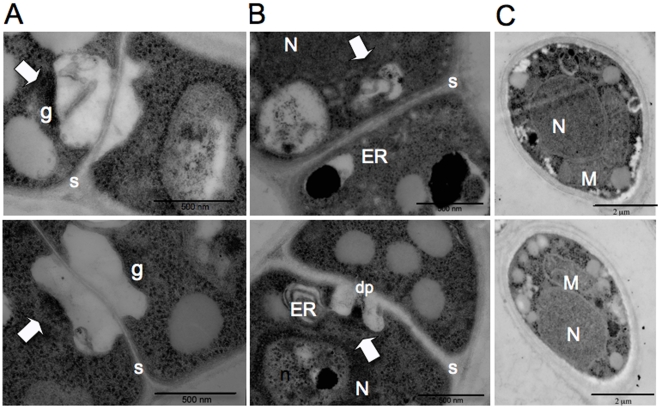
Transmission electron microscopy (TEM) of hyphal septa and basidiospores of *F. depauperata*. Panels A and B show different TEM images of the surface view of dolipore septa which is characteristic of basidiomycetes. Panel C shows TEM images from spores. The top row shows TEM images from strain CBS7855 and the bottom row from CBS7841. Arrows indicate electron dense occlusions and vesicles associated with the single septal opening. N denotes the nucleous, M the mitochondria, n the nucleolus, ER the endoplasmic reticulum, dp the dolipore, g the granular dolipore plug, and S the septa. Top panel shows strain CBS7855, and bottom panel shows CBS7841.

Surface field views from the colony edges were examined by scanning electron and light microscopy (Figures 3–4). These images confirmed the presence of four basipedal spore chains emerging from the terminally swollen basidia, features characteristic of the *Filobasidiella* clade (Figure 4). Under these conditions strain CBS7841 also showed more aerial hyphae, and longer intact spore chains, when compared to CBS7855 (Figures 3–5). Although isolate CBS7855 did not appear to have as many long spore chains as CBS7841, numerous dispersed and unattached spores were often found in close proximity to the spore chains of CBS7855. Further resolution of basidiospore shape was achieved through higher magnification SEM (Figure 4). Although the spore sizes of both strains are comparable (∼2×4 µm), the spore-shape differed. Strain CBS7855 produces circular and oval-shaped spores, while CBS7841 has circular, pentagonal, urn, or diamond-shaped spores (Figures 4–5). Fluorescent light microscopy allowed observation of the nuclear content of spores (Figure 5). A single nucleus is present in the basidiospores, which was confirmed by transmission electron microscopy (Figure 6). Fluorescent-activated cell sorting analysis (FACS) indicates that the spores appear haploid when compared to the reference strains of *C. neoformans* JEC21 (haploid) and XL1549 (diploid) (FACS results shown in supporting File S1). Under higher resolution SEM, the basidia at different stages of development showed a smooth surface texture in CBS7855, while a rougher surface is apparent in CBS7841 (Figure 4). These observed texture and spore morphology differences may be isolate- or species-specific. Alternatively, although the morphologies were reproducible among different replicates, they could also be artifacts of the fixation methods employed for SEM.

Nuclear content and septation of *F. depauperata* hyphae were examined by fluorescence microscopy and TEM. Images obtained by fluorescence microscopy reveal that *F. depauperata* hyphae are monokaryotic and lack clamp connections (Figure 5). A few hyphal cells of CBS7841 showed two irregular green spots located close together separated by a circular shaped dark region, which may represent the nucleolus. Since this observation could have also indicated the presence of two nuclei additional samples were stained with DAPI, which confirmed the presence of monokaryotic hyphae in CBS7841 (refer to supporting File S1).

TEM examination of septa morphology allowed visualization of the dolipore septation present in both strains of *F. depauperata* (Figure 6). Dolipore septa are a type of cross wall formed in vegetative hyphae, responsible for compartmentalizing the cells and restricting the movement of many organelles, such as nuclei, however allowing cytoplasmic exchanges. Dolipore septa consist of donut-shaped septal pore swellings often with a single central pore opening and sometimes with either vesicular or membranous parenthesomes. Analyses of characters associated with dolipore septation have aided the resolution of basidiomycete phylogeny [34]–[35]. The dolipore consist of and previous studies have shown the presence of dolipore septation without a septal cap or parenthosome in *C. neoformans* var. *neoformans* [36]. However, both strains of *F. depauperata*, CBS7841 and CBS7855, showed the presence vesicles and of electron dense material near the septal pore opening, in agreement with previous observations of CBS7841 [15].

### Synteny Analysis between *F. depauperata* and *C. neoformans*

To compare the genomic organization of *F. depauperata* and *C. neoformans* var. *neoformans*, a total of 7 fosmid clones (6B03, 5G13, 7G23, 7B24, 7D18, 8J20, and 8P15) from a genomic library of *F. depauperata* strain CBS7855 was sequenced, assembled and annotated. Four fosmids were selected for sequencing after probing the *F. depauperata* genomic library with several genes (*STE20, MYO2, LPD1, CID1*, and *STE11*) associated with the *MAT* locus of pathogenic *Cryptococcus* species. Fosmid 8P15 was positive for the *CID1* and *MYO2* genes, and fosmids 5G13 and 7D18 were positive for the *MYO2*, *STE20*, and *LPD1* genes. These two fosmids, 8P15 and 7D18, overlap covering 70 kb of contiguous sequence corresponding to regions of chromosome 4 where the *MAT* locus resides in *C. neoformans*. Fosmid 7B24 was positive for the *STE11* gene and this fosmid corresponds to regions on chromosomes 4 and 5 in *C. neoformans*. Three additional randomly selected fosmids were sequenced. Two of these fosmid clones (7G23 and 6B03) correspond to non-contiguous regions of chromosome 1, and the other fosmid clone (8J20) corresponds to a region of chromosome 12 in *C. neoformans* strain JEC21.

**Figure 7:**
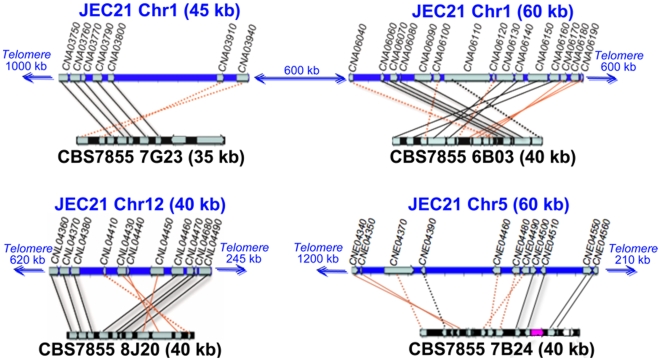
Genomic comparison between *C. neoformans* and *F. depauperata*. The gene order between *F. depauperata* and *C. neoformans* is conserved. Chromosomes of *C. neoformans* var. *neoformans* strain JEC21 are drawn in blue. Sequenced fosmids from the *F. depauperata* library of strain CBS7855 are drawn in black. Black lines connect genes in the same orientation, while red lines indicate inversions. Solid lines point to syntenic blocks where the adjacent gene order is conserved. Dotted lines connect orthologous genes where the adjacent genes are not conserved. Genes located on chromosomes 1, 5, and 12 of *C. neoformans* are shown in grey. The aim of this figure is to display the gene arrangements and directions. For simplicity purposes, only those genes present in both species, *C. neoformans* and *F. depauperata*, are shown.

The sequence from each fosmid (∼40 kb each) was analyzed by BLAST against the genome of *C. neoformans* in order to identify and annotate orthologous open reading frames (ORFs). A total of 66 ORFs identified in *F. depauperata* were present in *C. neoformans.* Annotated fosmid sequences were aligned using the genome of *C. neoformans* strain JEC21 as a reference in order to determine gene orientation and arrangement (Figure 7–9). The genomic regions from *F. depauperata* homologous to chromosomes 1, 5, and 12 of *C. neoformans* share several syntenic blocks in which 32 of the 44 genes in these regions displayed conserved gene order (score ≥1) while only 4 of the 22 genes mapping to chromosome 4 in *C. neoformans* were fully syntenic (Figure 7–9). The syntenic blocks can also be observed in the graphical representation of the synteny scores (Figure 9) based on conserved gene order (the scoring system disregards gene direction). This graph more clearly depicts that the lowest scoring region corresponded to chromosome 4 of *C. neoformans*, where the *MAT* locus resides.

**Figure 8 pone-0009620-g008:**
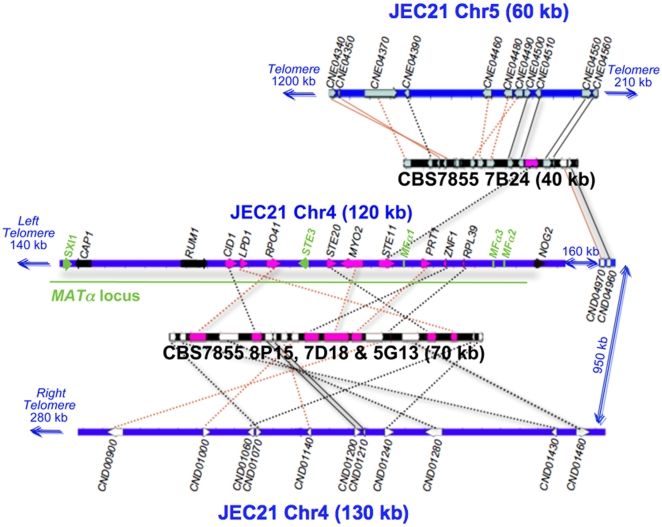
Genomic comparison of *MAT*-associated genes in *C. neoformans*. Several chromosomal translocations, fusions, and inversions appear be present in *F. depauperata* by comparison to the genome of *C. neoformans*. Chromosome 4 of *C. neoformans* var. *neoformans* strain JEC21 is drawn in blue. Sequenced fosmids from the *F. depauperata* library of strain CBS7855 are drawn in black. Black lines connect genes in the same orientation, while red lines indicate inversions. Solid lines point to syntenic blocks where the adjacent gene order is conserved. Dotted lines connect orthologous genes where the adjacent genes are not conserved. The aim of this figure is to display the gene arrangements and directions. For simplicity purposes, only those genes present in both species, *C. neoformans* and *F. depauperata*, are shown in grey, white, and pink. Genes located on chromosomes 5 and 12 of *C. neoformans* are shown in grey. Genes located on chromosome 4 of *C. neoformans* are shown in white while genes associated with the *MAT* locus of *Cryptococcus* are shown in pink. The α locus and the mating genes (the pheromone genes *MF*α, the pheromone receptor gene *STE3*, and the homeodomain transcription factor *SXI1*) of *C. neoformans* are shown in green. Genes shown in black, *CAP1* and *RUM1*, are associated with the *MAT* locus of *C. neoformans*, and *NOG2* flanks the *MAT* locus. These three genes were sequenced and identified in *F. depauperata*, however fosmids containing these genes were not found in the genomic library of *F. depauperata*.

**Figure 9:**
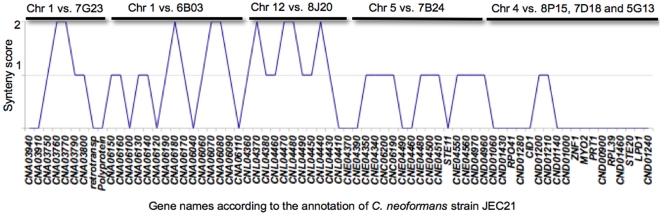
Graphical display of synteny between *F. depauperata* and *C. neoformans*. Summary of the synteny over the different genomic regions compared between *F. depauperata* and *C. neoformans*. The *x-*axis shows the gene names according to the annotations in the genome of *C. neoformans* strains JEC21. The *y-*axis shows the level of synteny. The maximum synteny score (score  = 2) was assigned to those ORFs fully syntenic because they are located between two adjacently conserved genes, while ORFs of adjacent pairs were assigned a lower score (score  = 1). Those ORFs with no adjacent genes conserved between the two species were given the lowest synteny score (score  = 0). The scoring system disregards the direction in which the genes are transcribed, and focuses on gene order by displaying the score for each gene calculated from the number of syntenic adjacent genes.

### Chromosomal Rearrangements

The minimum number of gene inversions, translocations and fusion event were predicted in the sequenced regions of *F. depauperata* using as a reference the genome of the sequenced strain of *C. neoformans*, JEC21 [37]. Figures 7 and 8 show the gene inversions (red solid lines connect inverted syntenic genes), and orthologous genes where the adjacent genes are not conserved (black and red dotted lines). The numbers of predicted chromosomal rearrangements, such gene inversions, chromosomal translocations and fusion event were determined by using GRIMM-Synteny software [29]–[30]. Predictions indicate that of the 22 ORFs of *F. depauperata* (fosmid clones 7G23 and 6B03) corresponding to regions of chromosome 1 in *C. neoformans* there were a minimum of 8 inversions. Likewise, of the 11 ORFs of *F. depauperata* (fosmid clone 8J20) corresponding to regions of chromosome 12 of *C. neoformans* there were a minimum of 2 predicted inversions. In contrast, of the 33 ORFs from *F. depauperata* (fosmid clones 7B24, 7D18, 5G13 and 8P15) that correspond to chromosomes 4 and 5, there were a minimum of 22 predicted inversions, 3 predicted translocations, and 2 predicted chromosomal fusion events. The total number of predicted chromosomal rearrangements in the regions mapping to chromosomes 4 and 5 are higher than those detected in chromosomes 1 and 12. The apparent translocation events near the “ancestral *MAT*” genes of *F. depauperata* are near the sub-telomeric regions of chromosome 4 (see Figure 8) and the translocated region of chromosome 5 appears to be in close proximity to the telomeric region of *C. neoformans* JEC21.

### Phylogeny of Genes Associated with the MAT Locus of *C. neoformans*

The *MAT* locus of *C. neoformans* is composed of >20 genes inherited as a single unit and located proximal to the telomere of chromosome 4. Nine of these 20 genes are found in the sequenced fosmids of *F. depauperata*. Although none of the 9 genes showed conserved synteny with either *MAT* allele in *C. neoformans*, 8 of these genes (*LPD1, CID1*, *RPO41, STE20, MYO2, PRT1, ZNF1*, and *RPL39*) are found in a contiguous (70 kb) contig in *F. depauperata* (Figure 8). By using degenerate primers we also obtained the sequences from *F. depauperata* for two additional genes associated with the *MAT* locus of *C. neoformans (CAP1* and *RUM1)*, and for one gene (*NOG2*) flanking the *MAT* locus of *C. neoformans.* The presence of these three genes in *F. depauperata* was confirmed by Southern hybridizations (data not shown) but we were unable to obtain clones positive for these genes in the fosmid library of *F. depauperata*.

**Figure 10:**
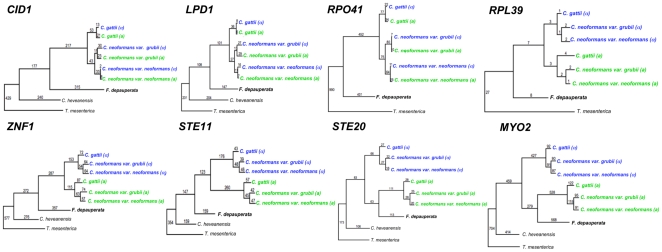
Phylogeny of genes linked to the *MAT* locus of *C. neoformans.* Two of the genes from *F. depauperata*, *STE20* and *MYO2,* hypothesized to be ancestrally acquired in the pheromone/pheromone receptor locus of the pathogenic *Cryptococcus* species, clustered with the *STE20* **a** and *MYO2* **a** alleles of *C. gattii* and *C. neoformans*. The remaining genes from *F. depauperata* exhibit a species-specific phylogeny. Genes from the *MAT* **a** alleles are shown in green, and genes from the *MAT*α allele are shown in blue. (α) indicates strains with the *MAT*α locus, and (a) indicates strains with the *MAT* **a** locus. The tree was constructed using Maximum Parsimony, and numbers indicate total changes in the branches. Statistical support was calculated from 1,000 bootstrap replicates. Bootstrap values were >70% in all branches (values not shown). Unrooted trees for Maximum Parsimony and Maximum likelihood are shown in supporting File S1.

Parsimony and maximum likelihood phylogenetic analyses suggest that several of the “ancestrally acquired genes” in the *MAT* **a** allele of *C. neoformans* and *C. gattii* have undergone a higher number of changes and substitutions/site than their respective counterparts linked to the *MAT*α allele. For example, the *STE20* **a** allele has 174 changes and 0.46 substitutions/site, while the *STE20*α allele shows 66 changes and 0.22 substitutions/site. The *STE11* **a** gene has 260 changes, and 0.66 substitutions/site, while the *STE11*α has 176 changes and 0.28 substitutions/site. Maximum parsimony trees displaying the number of changes for each branch are shown in Figure 10 (unrooted trees for Maximum Parsimony and Maximum Likelihood are shown in supporting File S1). However, the rest of the genes in these regions have an almost identical number of changes (Figure 10) and substitutions/site (data not shown).

Maximum Likelihood results showed the expected species phylogeny for all genes (supporting File S1). However, results from Maximum Parsimony analyses showed that the *STE20* and *MYO2* genes in *F. depauperata* formed a monophyletic cluster with *STE20*a and *MYO2*a alleles of *C. neoformans* and *C. gattii*. Furthermore, the *STE20* gene of *F. depauperata* shares 63 changes with the *STE20*a allele, and *MYO2* has 279 changes in common with the *MYO2*a allele of the pathogenic *Cryptococcus* species (number of changes shown above the branches in Figure 10). Collectively this data suggests that at least two of the *MAT*-associated genes in the pathogenic *Cryptococcus*, *STE20* and *MYO2*, may have been acquired into *MAT* prior to the divergence of *F. depauperata* (Figure 10). Although the *STE11* gene of *F. depauperata* shows the expected species-specific phylogeny, results from synteny plots revealed that this gene has modestly higher percent of DNA sequence identity to the *STE11*α allele (56%) than to the *STE11* **a** allele (50%) of *Cryptococcus* (data not shown). In contrast, the rest of the *MAT*-associated genes showed the same level of identity between the conserved regions of *F. depauperata* and either *MAT* allele of *C. neoformans* and *C. gattii*.

## Discussion

In the dimorphic basidiomycetous yeasts of the Tremellales, such as *C. neoformans* and *C. gattii*, the transition from yeast to filamentous growth is associated with the onset of sexual development. Recently, phylogenetic analyses revealed that the dimorphic human pathogenic *Cryptococcus* species, *C. neoformans* var. *grubii*, *C. neoformans* var. *neoformans*, and *C. gattii*, are closely related to three non-pathogenic species, *C. amylolentus*, *T. wingfieldii*, and *F. depauperata* [14]. Although these three non-pathogenic species are usually found as saprobes on decaying insects, their life cycle appears to be different. Two of the species, *C. amylolentus* and *T. wingfieldii* grow as yeasts, while *F. depauperata* grows exclusively as hyphae. Of the several environmental cues shown to impact the sexual cycle of the pathogenic *Cryptococcus* species [6]–[7], [20]–[21], [31]–[32] only pH appears to have an effect on fruiting in *F. depauperata*, (Figure 3). Nonetheless, in *F. depauperata* this environmental cue (low pH) is bypassed after prolonged incubation, suggesting that fruiting in *F. depauperata* might be under the control of different cues.

*F. depauperata*, *C. neoformans*, and *C. gattii* are classified as members of the *Filobasidiella* clade according to their basidial morphology Morphological examination of F. depauperata confirmed basidial features typical of the *Filobasidiales* (Figures 2–5). Higher resolution SEM enabled the observation of differences in basidia texture between both strains of *F. depauperata* (Figure 4, and supporting File S1). Interestingly, the strain of *F. depauperata* that displays a rough phenotype (CBS7841) also grows faster. In *C. neoformans* the transition from smooth to rough yeast colony morphology is triggered by cell density, and impacts cell survival and virulence [38]–[39]. Thus, we hypothesize that similar mechanisms could control the rough morphology of the basidia of *F. depauperata* CBS7841. Supporting this observation strain CBS7841 grows faster and might achieve a higher cell density earlier than the other strain, CBS7855. Alternatively, this texture difference could be specific to each strain. Similar differences in basidia texture have been described and employed for the morphological differentiation of *C. neoformans* (rough) and *C. gattii* (smooth) [13]. The other main morphological differences between the two strains are spore shape, and the maintenance of spore chain integrity (Figures 3–4). Because many of the spore chains of CBS7841 are intact, it is intriguing that higher magnification SEM also revealed that the spores of CBS7841 are often connected by a thin thread or spike-like structure (supporting File S1). This structure was not observed in CBS7855, and has not been previously described. Therefore it is possible that this spike-like spore connector is in part responsible for the structural maintenance of intact spore chains found only in isolate CBS7841. In summary examination of two strains of *F. depauperata* from different hosts and geographic origins, (CBS 7841 was isolated in Canada from a dead spider and CBS 7855 in the Czech Republic from a dead *Carpocapsa* caterpillar www.cbs.knaw.nl/yeast/BioloMICS.aspx), revealed clear morphological and molecular differences which suggest that these strains could potentially represent two populations or cryptic species of *F. depauperata* (Figure 1–2). Nonetheless, given the limited sample size (only two strains available) we are unable to determine whether these differences are strain-, population-, or species-specific.

Heterothallic mating in *C. neoformans* is characterized by the presence of dikaryotic filaments and fused clamp connections, while in self or homothallic mating (monokaryotic fruiting) the septated hyphal segments are monokaryotic and have unfused clamp connections [6], [9]. Previously, light microscopy and TEM examination of strain CBS7841 showed monokaryotic hyphae lack clamp connections and that synaptonemal complexes the presence of in the basidia of *F. depauperata* [15]–[16]. Thus, hyphae in *F. depauperata* resemble monokaryotic fruiting/same-sex mating in *C. neoformans*. Our results from fluorescence microscopy confirmed that both strains of *F. depauperata* lack clamp connections and contain a single nucleus per cell (Figures 4, and supporting File S1). Furthermore, our results from RAPD genotyping 39 progeny derived from CBS7855 showed the generation of at least one novel genotype (progeny #33, P33 Figure 2C–2D). This finding indicates that meiosis might be mutagenic and could serve as a mean to generate genetic diversity in *F. depauperata*.

Microscopic and morphological observations indicate that filamentation in *F. depauperata* resembles same-sex mating during monokaryotic fruiting, and therefore similar signaling cascades to those involved in monokaryotic fruiting could be present in *F. depauperata*. In the case of *F. depauperata* compared to *C. neoformans* and *C. gattii*, the filamentous hyphal growth mode appears to correspond to the sexual stage of the yeast species. *F. depauperata* is not only homothallic, but an obligately sexual organism, permanently undergoing sexual reproduction which is likely energetically costly. Consequently, we hypothesize that strong adaptive benefits, in addition to the production of progeny and new genotypes (Figure 2), might be provided by this mode of growth. For example, slow-growing fungi are often found in extreme habitats because they tend to be more tolerant to stressful, harsh, and unfavorable conditions [40]–[42]. Perhaps *F. depauperata* is also constantly engaged in sexual reproduction because its slow growth contributes to its ability to grow in the presence of several antimicrobial compounds (supporting File S1) known to be toxic to *C. neoformans*, *C. gattii*, and other fungi.

Sexual reproduction in *C. neoformans* and *C. gattii* is controlled by a bipolar system in which a single mating type (*MAT*) locus specifies cell type identity. The *MAT* locus in *C. neoformans* and *C. gattii*, which is hypothesized to have evolved from a common ancestral tetrapolar locus, shares several features reminiscent of sex chromosomes in multi-cellular eukaryotes. They are unusually large (>100 kb) when compared to most fungi [2] and recombination is suppressed within the *MAT* locus during meiosis and activated adjacent to it [43]. The *MAT* loci of *C. neoformans* and *C. gattii* encodes >20 genes and numerous repetitive and transposable elements that are inherited as a single contiguous locus [2], [23], [43]. Genes within the *MAT* loci are highly rearranged among the *Cryptococcus* pathogenic species. Fifteen of these genes (such as *STE11, STE20, MYO2, CAP1, RPL39, PRT1*, and *ZNF1*) display a mating type specific phylogeny consistent with an ancestral association with *MAT.* The five other genes (*LPD1, CID1, RPO41, BSP2*, and *GEF1*) are syntenic and display species-specific phylogeny [2]. In *F. depauperata*, three of these apparently species-specific genes (*LPD1, CID1*, and *RPO41*), and five of the ancestral *MAT* associated genes (*STE20, MYO2, PRT1, ZNF1*, and *RPL39*) are found in a contiguous cluster (Figure 8). Thus, at least three of the five recently acquired genes were in close proximity in the common ancestor of *Cryptococcus* and *F*. *depauperata*. The pheromone and pheromone receptor genes involved in the recognition of a compatible mating partner in the genomes of the heterothallic close relatives, *C. neoformans*, *C. gattii*, *C. heveanensis*, and *T. mesenterica* are arranged in a cluster that includes *STE20, PRT1, ZNF1, RPL39*, and *STE11*, indicating that this gene cluster is ancestral to their species divergence and that outcrossing is the ancestral mode of reproduction [2], [23]. Unlike in *C. neoformans* and *C. gattii* transposable elements and repeats were not found within these regions in *F. depauperata*, suggesting that this elements may have been incorporated into this region specifically in the lineage of the pathogenic *Cryptococcus* species. Furthermore, since in *C. neoformans* and *C. gattii* the *STE11*a, *STE20*a, *and MYO2*a alleles appear to have a higher number of changes and substitutions than their α counterparts (Figure 10), it is possible that the ancestral *MAT* **a** locus underwent accelerated evolution prior to the divergence of the pathogenic *Cryptococcus*. Although we sequenced several fosmids from *F. depauperata* that contained the genes linked to *MAT* in the pathogenic *Cryptococcus* species, we did not find any of the sex determining genes in *F. depauperata* (Figure 8) in this region. Therefore, the pheromone and pheromone receptor genes in *F. depauperata* could be located in a different genomic region or could be missing from the genome altogether. Nonetheless, sequencing the whole genome maybe necessary to thoroughly examine the presence of sex determining genes and repetitive elements in *F. depauperata*.

In the fungal kingdom homothallism is present in three of the main fungal lineages, zygomycetes and ascomycetes, and less often in basidiomycetes. Five main mechanisms for homothallism have been characterized in fungi: a) same-sex mating (monokaryotic fruiting), b) fusion of compatible mating type genes into one locus, c) unlinked compatible mating types genes present in one genome, d) mating-type switching, or e) packaging of two compatible nuclei into one spore (pseudohomothallism) [44]. Although we were unable to characterize the molecular nature of homothallism in *F. depauperata*, we propose that the evidence provided in the current study supports monokaryotic fruiting, or the presence of either linked or unlinked compatible mating types genes into one genome, as the most likely models for homothallism in *F. depauperata*. Furthermore, we also put forward the existence of an alternative model in which even in the absence of mating type genes epistatic or genetic changes altering the regulation of the downstream components of the cascades controlling mating and filamentation and could result in the observed life cycle of *F. depauperata* (supporting File S1). For example, the whole genome sequence analysis of the homothallic yeast *Lodderomyces elongisporus*, a close relative of *Candida parapsilosis* and *Candida albicans*, revealed that this species lacks the mating type locus cell identity genes (**a**1, **a**2, α1, α2) [45]. Further studies will be required to establish whether this is truly sexual reproduction occurring in the absence of the canonical *MAT*, but if so, it would establish a novel paradigm that might apply to other homothallic species, such as *F. depauperata*.

Although Likelihood analyses showed the expected species-specific phylogeny for all genes (supporting File S1), parsimony analyses suggest that at least two of the *MAT*-associated genes in the pathogenic *Cryptococcus*, *STE20* and *MYO2*, may have been acquired into *MAT* prior to the divergence of *F. depauperata* (Figure 10). Therefore, it is possible that a single mating type allele might be present in the genome of *F. depauperata*, which could explain the resemblance of its life cycle to monokaryotic fruiting in *C. neoformans*. The *STE11* gene in *F. depauperata* does not appear to be mating type specific by either Parsimony or Maximum Likelihood analyses (Figure 10). However, results from DNA identity plots suggest that the *STE11* gene shares higher identity to the *STE11*α allele of *C. neoformans*. Although the difference in shared identity might not be statistically significant, this finding prompted us to discuss the idea that the homothallic sexual cycle of *F. depauperata* could be the result of compatible mating type loci (*MAT* **a** and *MAT*α) present in one nucleus. Interestingly, several studies have shown in *C. neoformans* that haploid strains engineered to contain both compatible homeodomain genes become self-fertile [12], [46]–[47]. Furthermore, these strains of *C. neoformans* can also fruit and form hyphae with unfused clamp connections [12], a process that also resembles monokaryotic hyphae. Thus, the presence of two compatible mating alleles in different genomic locations could also result in self-fertility in *F. depauperata*. Such is the case for the homothallic fungi *Aspergillus nidulans* and *Neosartorya fischeri* where compatible mating genes are present in different chromosomal locations, or unlinked but the same chromosome [48]–[50].

Alternatively, similar to the majority of the homothallic fungi in the ascomycetes, *F. depauperata* might contain both mating alleles which are fused or are linked in one locus. For example, in the genus *Cochliobolus* while several species in this genus are outcrossing, most of the homothallic species in this genus contain either fused or linked compatible mating genes [51]. Additional examples of fused compatible mating loci are also present in the homothallic fungi in the genus *Sordaria* and *Fusarium* [52]–[53]. These studies in ascomycetous fungi suggest that transitions between heterothallic and homothallic modes of reproduction usually involve recombination and chromosomal translocations of the regions flanking their *MAT* loci. In *C. neoformans* it has been proposed that the resolution of an intermediate tripolar locus resulted in the bipolar locus we observed today [2], [12]. The resolution of this transitional tripolar intermediate might also result in a strain that inherits compatible alleles and is therefore homothallic. In such a scenario the compatible alleles might subsequently undergo additional rearrangement through nonreciprocal recombination and chromosomal translocation events to create a stable homothallic strain or species.

In *C*. *neoformans* and *C. gattii*, even under optimal laboratory conditions for mating and monokaryotic fruiting large amounts of yeast cells are always observed, therefore the complete absence of a yeast stage in *F. depauperata* supports the hypothesis that the signaling cascade for filamentation is regulated differently in this fungus. Thus, we propose that the filamentous life style in *F. depauperata* could be the result of an alteration of downstream signaling components in the filamentation pathway. Since this pathway is usually activated in response to the presence of a compatible mating partner, the need for mating type genes involved in pheromone recognition and homeodomain protein compatibility could be bypassed, and eventually the pheromone/pheromone receptor genes might even be lost from the genome. The relationship between the pathogenic yeast species, *C. neoformans* and *C. gattii*, and the filamentous fungus *F. depauperata* is similar to the relationship between the model budding yeast *S. cerevisiae* and the closely related species *Ashbya gossypii*, which grows exclusively as a filamentous fungus. Whole genome analysis of *A. gossypii* reveals that 97% of the genes are shared between the two species, and extensive mutagenesis studies of many of the few (∼3%) novel genes has not revealed obvious candidates that explain the marked disparity in growth morphologies [54]. Thus, it is also likely that more subtle changes in gene function or regulation underlie their morphological differences.

Synteny analyses of five genomic loci revealed that the estimated number of translocations in *F. depauperata* was higher in regions mapping to chromosomes 4 and 5 of *C. neoformans*. One of the translocated regions in *F. depauperata* involves the contig containing the *STE11* gene (contig 7B24; Figures 7 and 8). Although this gene is also found as part of a cluster of genes associated with the pheromone receptor in other closely related fungi (*C. neoformans, C. gattii, C. heveanensis*, and *T. mesenterica*), in *F. depauperata* the *STE11* gene was translocated outside the cluster. Previous studies have shown that *STE11* gene product acts in the MAPKK cascades involved in filamentation and mating in many fungi [55]–[56]. Deletion of the *STE11*α gene in *C. neoformans* resulted in sterility and filamentation defects during monokaryotic fruiting [33], [57]–[58]. Interestingly, later on Lengeler et al. 2002 also found that an ∼4 kb insertion took place between the pheromone gene and the *STE11α* in at least one strain of *C. neoformans*, indicating that regions around *STE11* gene might also be prone to rearrangements in these fungi, and not only in *F. depauperata*. The translocation of the *STE11* gene in *F. depauperata* might partially explain the missing pheromone genes in *F. depauperata* since these genes could have been moved to a different location. Furthermore, the translocation of *STE11* may also alter its regulation, expression, and/or protein activity. Such types of changes in the signaling cascade controlling hyphal growth could potentially explain the ability of *F. depauperata* to filament and fruit regardless of environmental cues, such as light, media, and pH.

Furthermore, synteny analyses revealed that the contig containing the *STE11* gene also has genes found in close proximity to a 40 kb (sub-telomeric) region of chromosome 4 of *C. neoformans* (Figures 7–8). This 40 kb region has been associated with sub-telomeric rearrangements and an intervarietal introgression between chromosomes 4 and 5 in *C. neoformans* var. *neoformans* and var. *grubii* [59]. Sub-telomeric rearrangements involving both arms of chromosome 4 also appear to be present in the *MAT* locus of *F. depauperata* (Figures 7–8) and *C. heveanensis* (Metin et al. *in prep.*). *F. depauperata* and *C. heveanensis* share at least nine *MAT*-associated genes, and they also share one gene (CND01240) found in the sub-telomeric region of JEC21. These findings suggest that at least one ancestral sub-telomeric intra-chromosomal rearrangement shaped the evolution of chromosome 4, and thereby the *MAT* locus in these fungi. Interestingly, reciprocal chromosomal translocations and chromosomal fusion events have also been previously identified in chromosomes 8, 9, and 12 in *C. neoformans* var. *neoformans* [60]–[61] and chromosomal rearrangements have been shown to occur in *C. neoformans* during infection of mammalian hosts [62]–[63]. Therefore, intra- and inter-chromosomal rearrangement events, predominantly those resulting in gene losses and acquisitions in this region, appear to be driving sexual divergence, speciation, and adaptation in the *Filobasidiales* (particularly in the human pathogenic *Cryptococcus* species).

It is well accepted that chromosomal rearrangements vary within chromosomal position in eukaryotes, for example sub-telomeric regions have been shown to be more susceptible to translocations, insertions, and deletions. Furthermore, the rate of chromosomal rearrangements has also been shown to vary between chromosomes; for example, autosomes and sex chromosomes of the same organism display different rates [64]. The results from synteny studies between *F. depauperata* and *C. neoformans* indicated that an increased number of chromosomal rearrangements have taken place in the genes associated with the *MAT* locus in *C. neoformans*, while synteny is highly conserved in the other genomic regions analyzed (Figures 7–9). Given the genomic plasticity of many eukaryotes, chromosomal events contribute to speciation only if they are fixed and confer an adaptive advantage [65]. Although the genomes of *C. neoformans* var. *neoformans* and *grubii* are mostly collinear and share ∼85–95% DNA identity, several inversions and translocations, including those within *MAT*, may have resulted in a genetic barrier, effectively making the two varieties different crossing populations and species [59]. Genomic comparisons that include the two *C. neoformans* varieties and the genome of two *C. gattii* molecular groups are currently in progress (Kronstad et al. in prep.). The current study sets the stage for future complete genomic comparisons between the pathogenic *Cryptococcus* species and their closest saprobic relatives. Since most human pathogenic fungi are dimorphic, with yeast and filamentous stages of growth, these types of genomic studies will provide further insight into genomic events and selective forces that shape chromosome evolution and impact mating, morphogenesis, pathogenicity and habitat speciation in fungi and other eukaryotes.

## Supporting Information

File S1 This file contains supplementary figures with legends.
(8.32 MB DOC)
