# Supplementary material for: Morphological and Genomic Characterization of Filobasidiella depauperata: A Homothallic Sibling Species of the Pathogenic Cryptococcus Species Complex
Source: PLoS One. 2010 Mar 10;5(3):e9620. doi: 10.1371/journal.pone.0009620 (PMC2835752; doi:10.1371/journal.pone.0009620)

**Supporting File S1**

**List of random primers used for RAPD genotyping**

This table shows the name of the primers, sequences and PCR conditions under which the different primers were used. The results obtained for CBS7841, CBS7855, and 39 progeny derived from CBS7855 are summarized in the last two columns. Yes indicates differential bands identified.

**
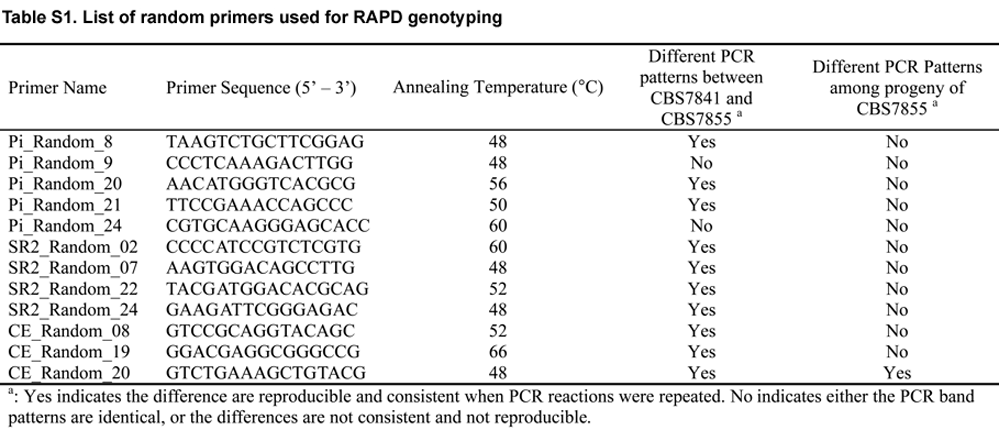
**

**DNA sequence divergency between both strains of *F. depauperata.***

Pairwise alignment shows 2% sequence divergence. The differences in the sequences between both strains of *F. depauperata* are transitional changes at the nucleotide level. Sequences of both strains of *F. depauperata* were aligned and concatenated in MacClade for pairwise comparison.

**
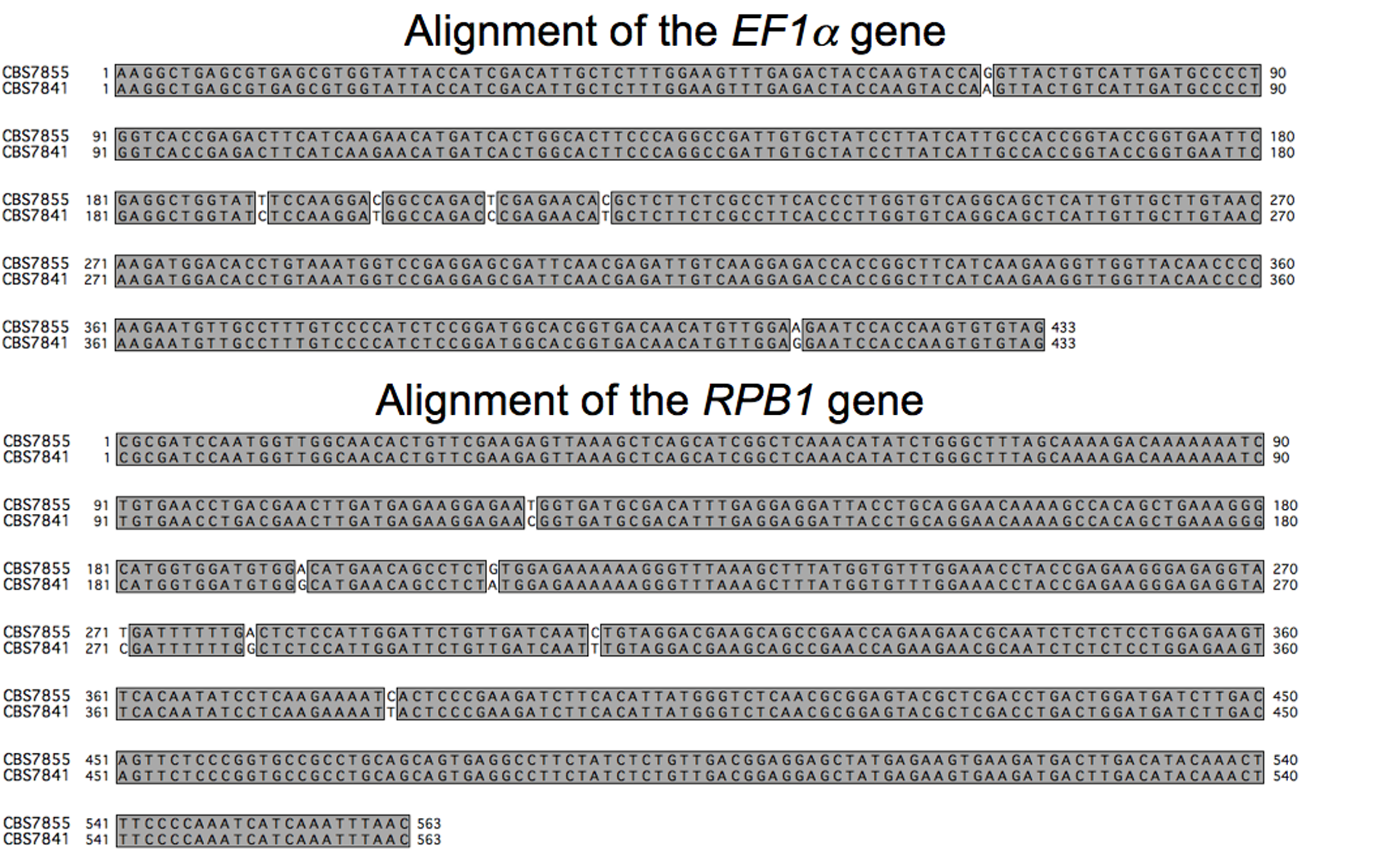
**

**Phylogeny of *F. depauperata.***

Maximum Likelihood and Maximum Parsimony unrooted trees displaying the phylogeny of both strains of *F. depauperata*. (α) indicates strains with the *MAT*α locus. Statistical support was calculated from 1,000 bootstrap replicates. Bootstrap values were > 70% in all branches (values not shown).

**
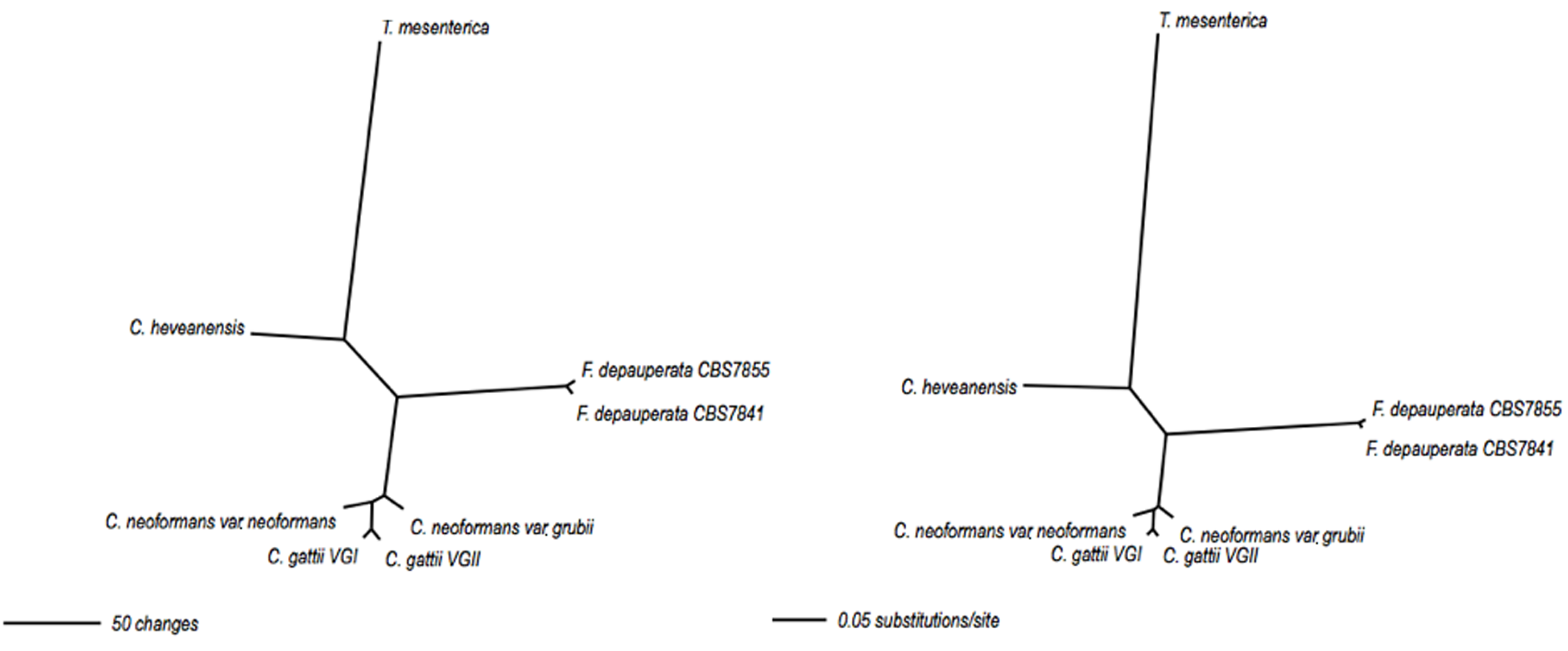
**

**Colony growth morphology differs between the two strains of *F. depauperata.***

Growth of both strains of *F. depauperata* in the presence of different antimicrobial compounds is shown. Strain CBS7841 grows faster than strain CBS7855. A total of 10^5^  and 10^4^  spores from each strain were inoculated onto rich agar media (Yeast Peptone Dextrose, YPD), and also rich media with antimicrobial compounds (NEO = neomycin 100 μg/ml, NAT = nourseothricin 100 μg/ml, and HYG = hygromycin 200 μg/ml). Top panel shows strain CBS7841 and the bottom panel shows CBS7855. Photographs were taken after 7 days (7d) or 14 days (14d).

**
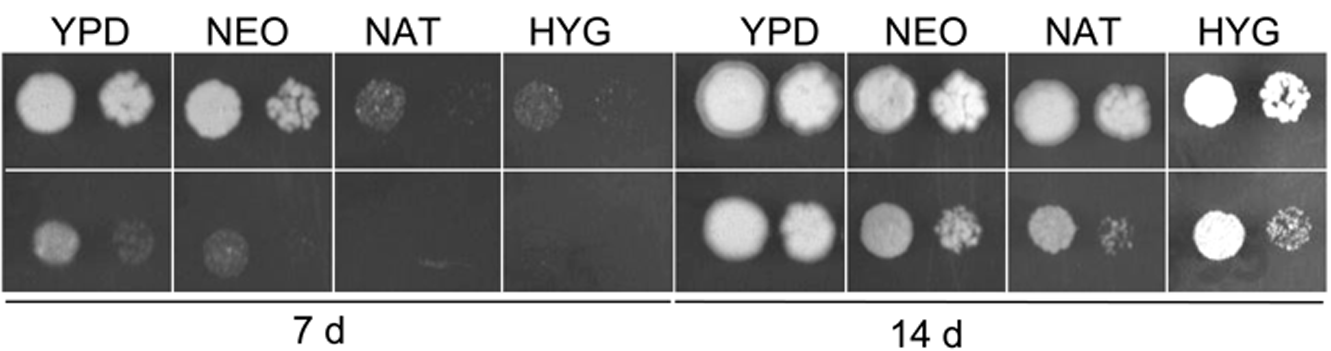
**

**Life cycle of *F. depauperata* strain CBS7841.**

Spores from strains CBS7841 were germinated on slides coated with YPD medium. Growth was monitored periodically and photographs were taken to document the life cycle of *F. depauperata*. Samples were stained with sytox green and calcoflour white to visualize nucleic acids and chitin in the cell walls during fluorescence microscopy.

**
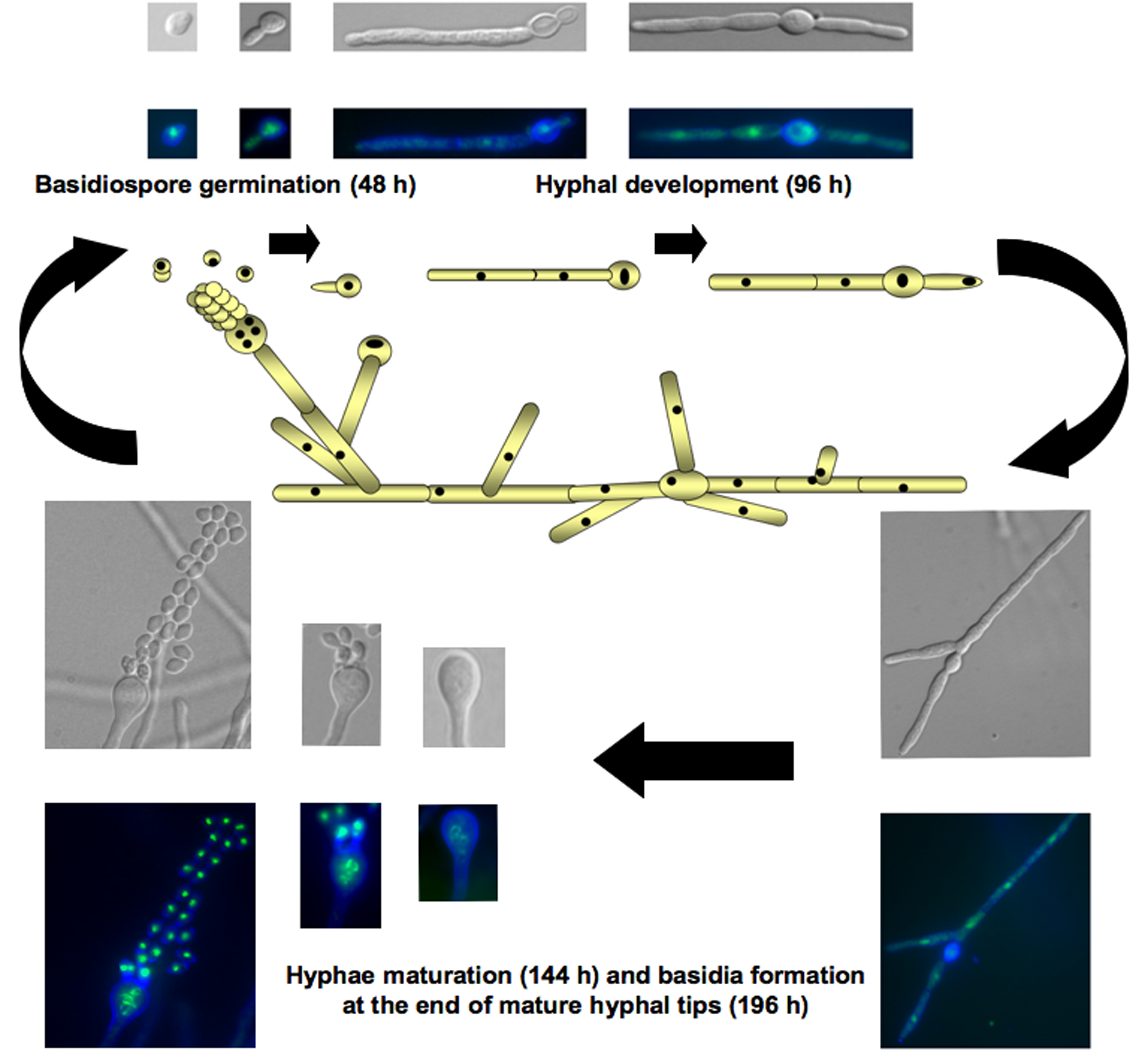
**

**Fluorescent-activated cell sorting analysis (FACS) of basidiospores from *F. depauperata.***

Basidiospores from *F. depauperata* have equal amount of nuclear content to those of haploid yeast cells from *C. neoformans* (~20 Mb) based on Fluorescence-activated cell sorter (FACS) analysis. Diploid and haploid yeast cells from *C. neoformans*, and basidiospores from both strains of *F. depauperata* were diluted and subjected to FACS analysis. Strain XL1549 is a diploid control, and JEC21 a haploid control. The control shows two peaks, one in MI representing cells non-dividing (G1), and one in M2 showing the relative amounts of dividing cells (G2). The vertical axis shows the number of cells, and the horizontal axis the relative DNA content. >10,000 cells of each strain were analyzed. DNA content was determined by relative fluorescence of stained genomic DNA for the haploid and diploid controls of *C. neoformans* strains*.*

**
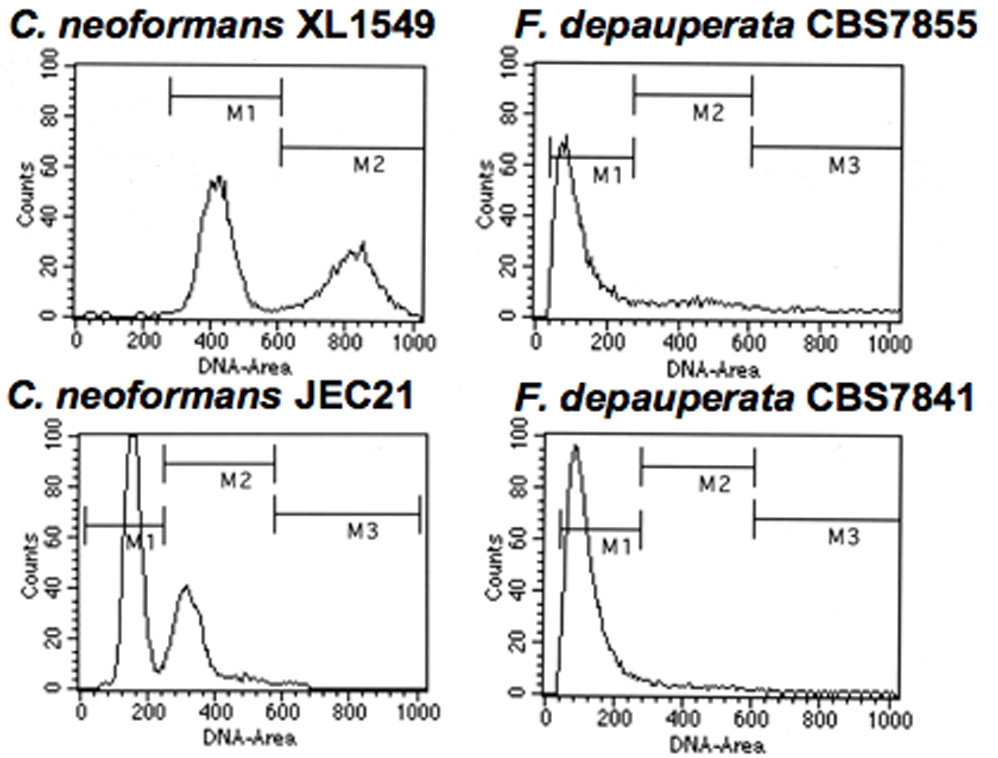
**

**High resolution SEM of basidia and basidiospores.**

Arrows indicate the spike-like structure identified in strain CBS7855. Bar = 5 μm.

**
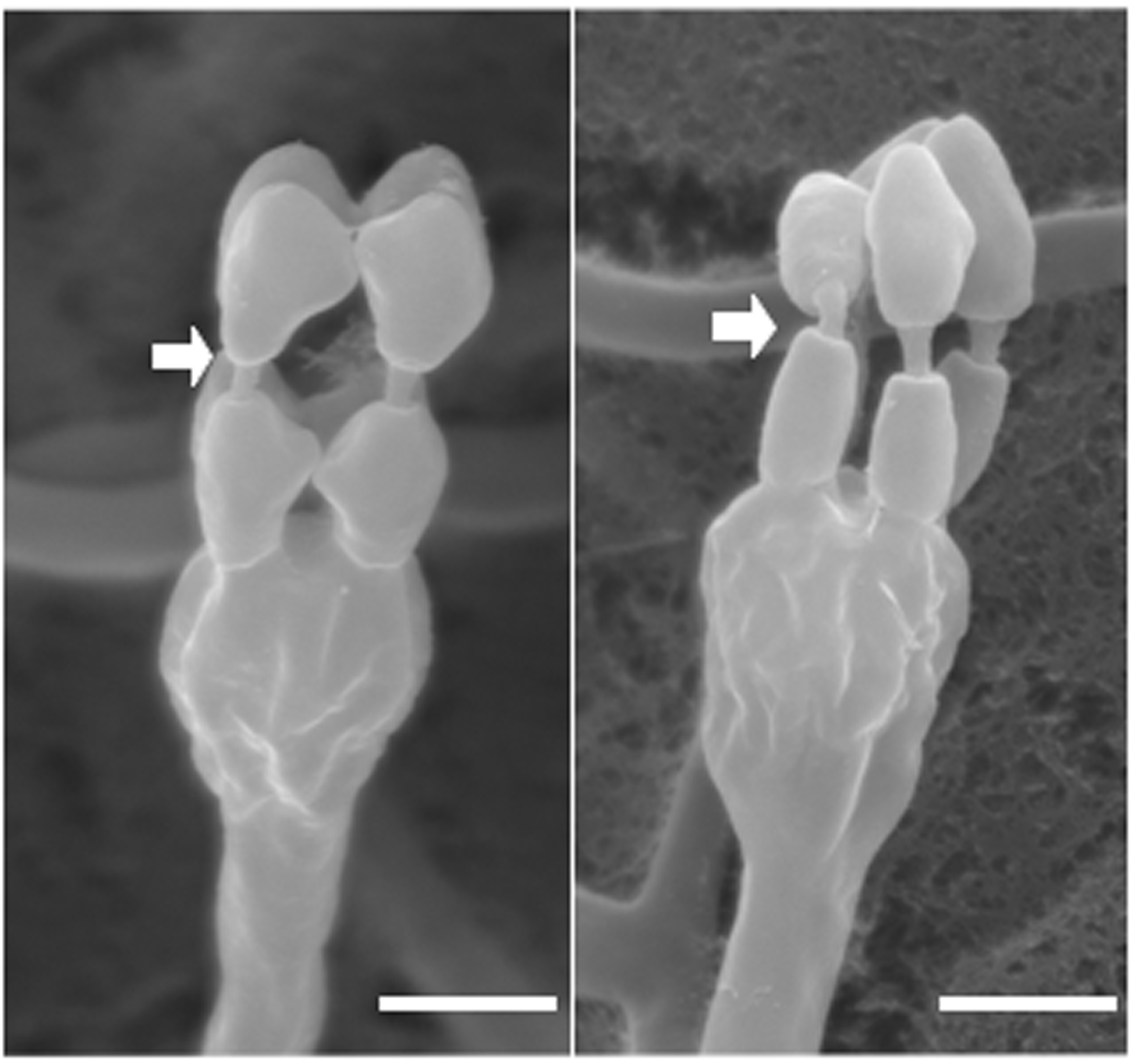
**

**Nuclear content of basidia, basidiospores, and hyphae of strain CBS7841.**

To confirm nuclear content samples were subjected to further staining of nucleic acids with DAPI (in white). DIC image is shown on the left and fluorescent light microscopy images are shown on the right, Bar = 20 μm.

**
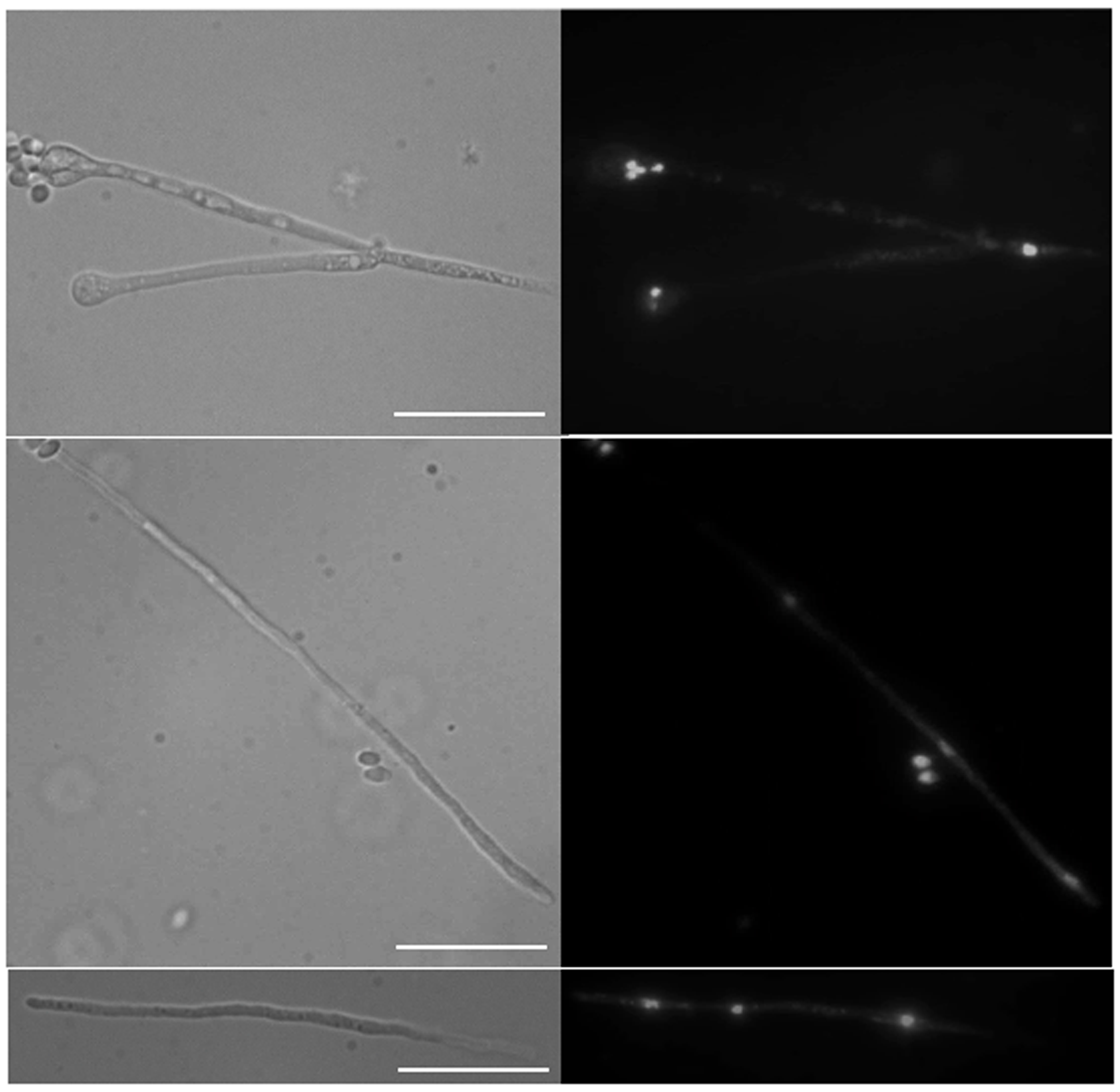
**

**Unrooted Maximum Parsimony trees of genes from *F. depauperata* that are linked to the *MAT* locus of *C. neoformans.***

To differentiate both alleles, genes from the *MAT*α allele are shown in blue. (α) indicates strains with the *MAT*α locus, and (a) indicates strains with the *MAT***a** locus. Statistical support was calculated from 1,000 bootstrap replicates. Bootstrap values were > 70% in all branches (values not shown).


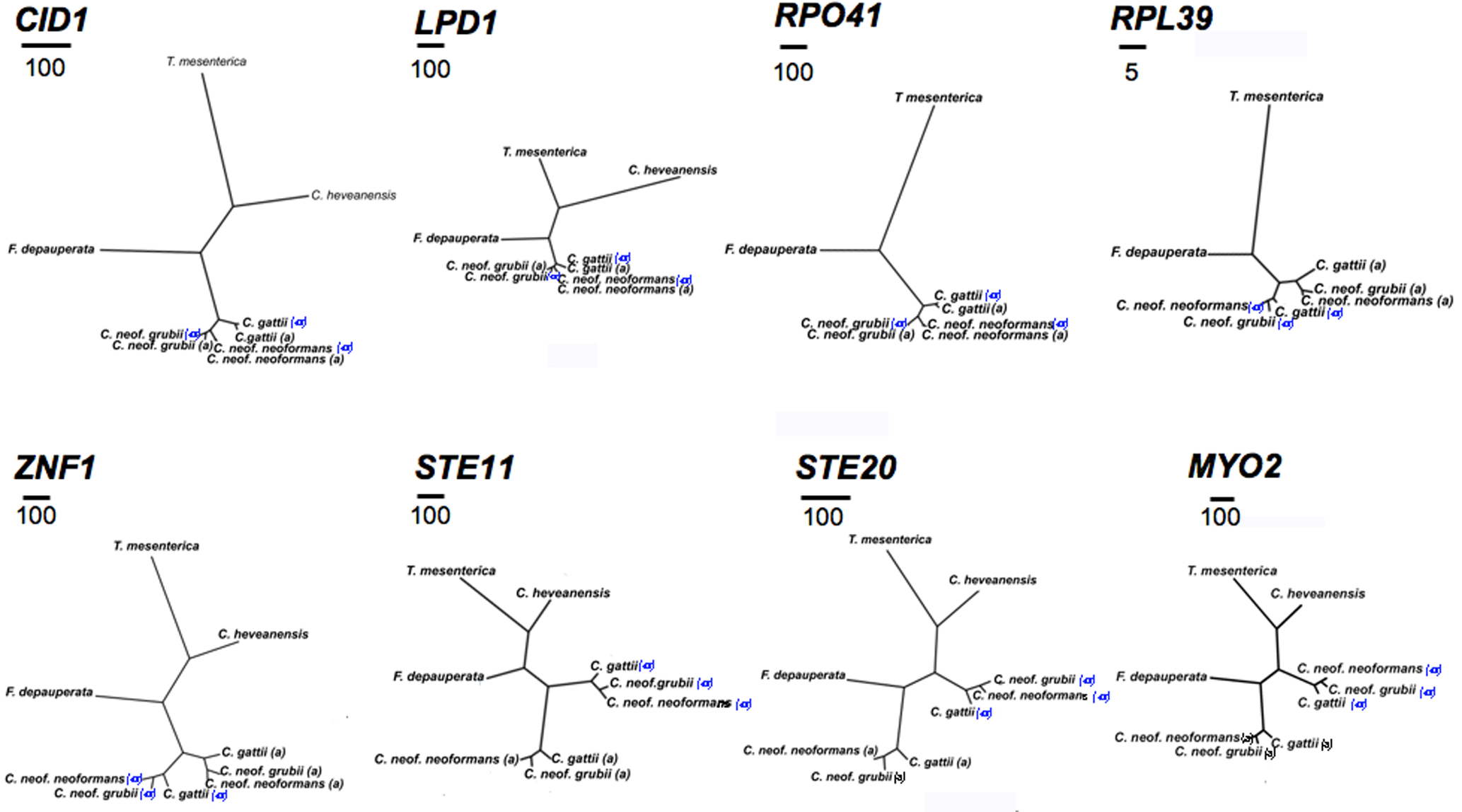


**Unrooted Maximum Likelihood trees of genes from *F. depauperata* that are linked to the *MAT* locus of *C. neoformans.***

To differentiate both alleles, genes from the *MAT*α allele are shown in blue. (α) indicates strains with the *MAT*α locus, and “a” indicates strains with the *MAT***a** locus. Statistical support was calculated from 1,000 bootstrap replicates. Bootstrap values were > 70% in all branches (values not shown) except for those within the pathogenic *Cryptococcus* species (*C. gattii, C. neoformans* var. *neoformans and C. neoformans* var. *grubii* branches had <65% bootstrap support).


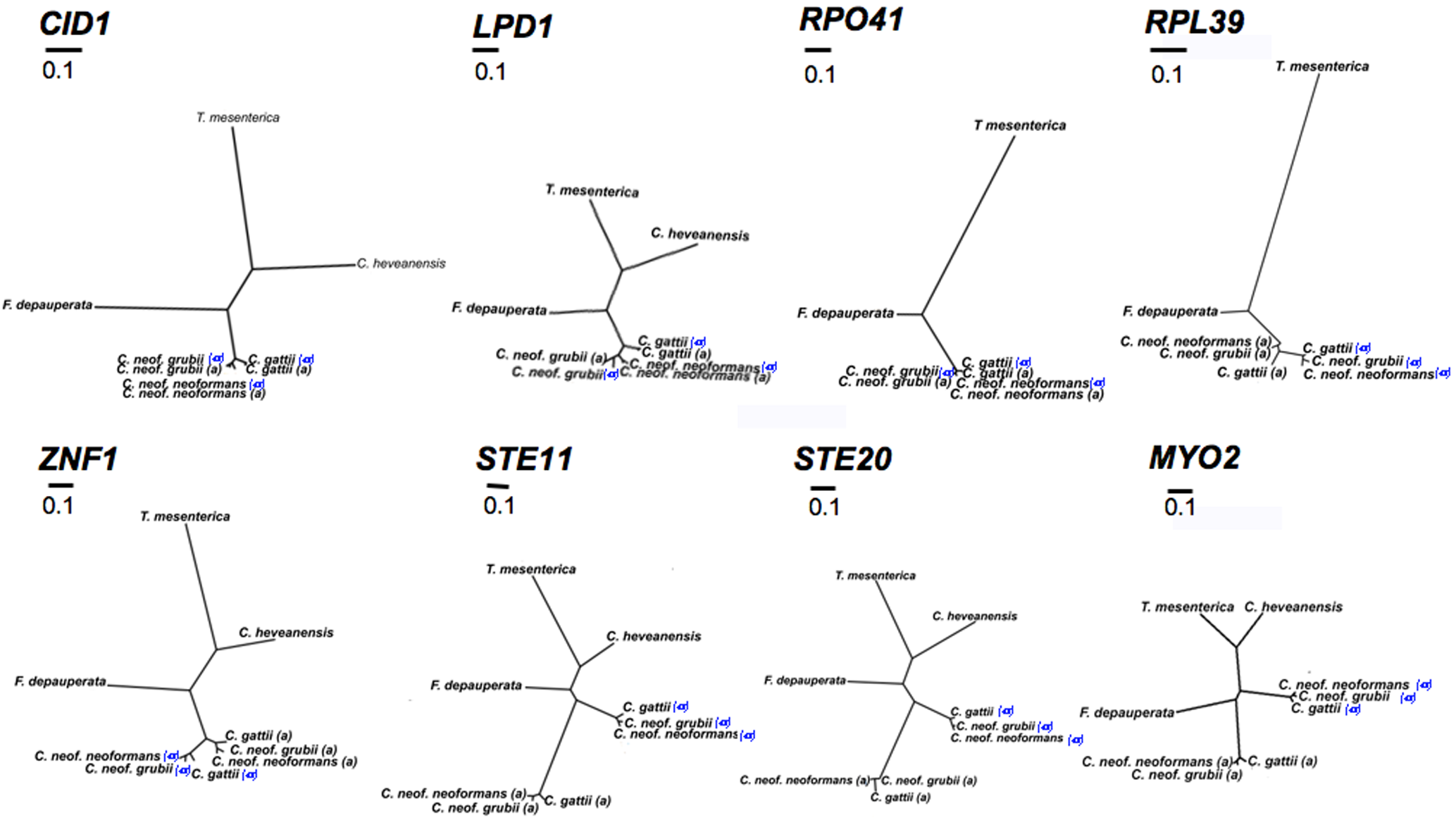

Supplement: File S1 — This file contains supplementary figures with legends. (8.32 MB DOC) [file pone.0009620.s001.doc]
